# Mechanism-aware and safety-validated AI for drug repurposing: a critical review and translational pharmacology framework

**DOI:** 10.3389/fphar.2026.1915973

**Published:** 2026-09-18

**Authors:** Moumita Hazra, Harishchander Anandaram

**Affiliations:** 1 Department of Pharmacology, Maharajah’s Institute of Medical Sciences and Hospital, Dr. N. T. R. University of Health Sciences, Dr. Y. S. R. University of Health Sciences, Andhra Pradesh, India; 2 Amrita School of Artificial Intelligence, Coimbatore, Amrita Vishwa Vidyapeetham, Coimbatore, Tamil Nadu, India

**Keywords:** artificial intelligence, drug repurposing, mechanism-aware AI, pharmacovigilance, safety validation, translational pharmacology

## Abstract

**Background:**

Artificial intelligence (AI)-assisted drug repurposing seeks to identify new therapeutic applications for existing drugs. Nevertheless, most of the existing studies are centered on prediction scores and candidate ranking, and there is still relatively little attention paid to the pharmacological mechanisms, safety and translational validation.

**Objective:**

This critical review summarizes existing AI approaches to drug repurposing, identifies their mechanistic, safety, and translational barriers, and incorporates the emerging evidence gaps into a mechanism-driven and safety-proven translational pharmacology model.

**Findings:**

The literature reviewed shows that machine learning, deep learning, network medicine, knowledge graphs, graph neural networks, and foundation models have the potential to significantly increase the number of candidates identified, but they have marked variations in terms of interpretability, biological support, safety integration, external validation, and clinical applicability. In all of these strategies, common shortcomings are inadequate mechanism validation, poor causal evidence, incomplete dose and safety evaluation, weak pharmacovigilance integration, and reliance on internal validation, and varying experimental or real-world confirmatory evidence. These persistent evidence gaps were incorporated into a sequence of steps that incorporates candidate predictions, mechanism mapping, determination of causal plausibility, safety and toxicity evaluation, pharmacovigilance, real-world validation, expert pharmacology review and evidence-based candidate prioritization.

**Conclusion:**

AI-driven drug repurposing presents huge potential in the field of therapeutic innovation, but current applications require more clinical evidence and pharmacological mechanisms demonstrating safety, as well as clinical relevance. The proposed framework can support the more reliable prioritization of candidates for experimental testing, real world validation and future clinical testing.

## 1 Introduction

### 1.1 The shift from conventional discovery to AI-enabled repurposing

Drug discovery remains an expensive, lengthy, and high-risk process, with many candidates failing because of inadequate efficacy, safety, or pharmacokinetic properties. These limitations have increased interest in drug repurposing, the process of repurposing existing drugs for new therapeutic applications. In addition, repurposed drugs may offer a quicker and potentially safer alternative to *de novo* drug development, as there is prior information available regarding the pharmacology, dosage, pharmacokinetics, toxicity, and safety of the drug (Pu et al., 2019; Talevi and Bellera, 2020). This strategy has been further extended by artificial intelligence, which allows for large-scale analysis of chemical structures, drug–target interactions, disease genes, gene expression profiles, biomedical literature, clinical records and adverse-event data. AI capabilities enable the discovery of potential drug–disease relationships that might not be apparent from traditional experiments or clinical observations (Vamathevan et al., 2019; Mak et al., 2024).

### 1.2 Why AI-driven repurposing is pharmacologically attractive

A major advantage of AI-assisted drug repurposing is its capacity to integrate multiple layers of biomedical information within a unified analytical framework. AI models can connect drug–target interactions, disease-associated genes, omics signatures, chemical structures, electronic health records, biomedical literature and safety databases to reveal complex drug–pathway–disease phenotype–subgroup relationships (Wan et al., 2025; Singh et al., 2023). This field has recently been strengthened by the advancement of network medicine, biomedical knowledge graphs, graph neural networks and foundation models, which provide more structured and interpretable connections between drugs and diseases. In particular, Knowledge graph-based approaches can follow explanatory links between drugs, targets, pathways, phenotypes, diseases and adverse events (Perdomo-Quinteiro and Belmonte-Hernández, 2024). Beyond just numerical predictions, the ability of AI to use mechanism-oriented frameworks like KGML-xDTD, path-based graph completion methods, and graph foundation models to inform biologically meaningful repurposing hypotheses further illustrates how AI can help generate repurposing ideas (Huaneg et al., 2024; Jiménez et al., 2024; Ma et al., 2023).

### 1.3 The core gap: Prediction is not pharmacological evidence

Although AI has substantially expanded drug-repurposing candidate generation, computational ranking alone does not establish therapeutic plausibility (Liu et al., 2021; Yan et al., 2024). This review therefore examines the evidence required to progress from candidate prediction to translational assessment, with particular attention to mechanistic and causal support, indication-specific safety, and independent experimental or clinical validation. These evidence domains are examined separately in the following sections and subsequently integrated into the proposed translational framework.

## 2 Review methodology and scope

### 2.1 Review design and objectives

This article adopts a structured critical narrative-review approach to examine how artificial intelligence is currently used in drug repurposing and, more specifically, how computational predictions are supported by mechanistic, pharmacological, safety, experimental, and clinical evidence. The review was organized around three questions: (i) what major AI methodologies are currently used for drug-repurposing candidate identification; (ii) what limitations affect their mechanistic interpretability, safety assessment, reproducibility, and translational validity; and (iii) what evidence is required to progress an AI-generated candidate from computational prioritization toward experimental or clinical evaluation. The translational framework presented later in the review was developed as a synthesis of these recurring evidence requirements rather than being treated as an independent starting proposition.

### 2.2 Structured literature search strategy

A systematic and repeatable literature search was carried out to locate studies relevant to drug repurposing using artificial intelligence (AI) and mechanistic, safety, and translational validation studies. Literature was searched in various databases, including PubMed/MEDLINE, Scopus, Web of Science Core Collection and Embase. The search included literature related to drug repurposing and AI/ML/deep learning, knowledge graphs, graph NN, foundation models, mechanistic validation, pharmacovigilance, pharmacological safety, RWE, and translation assessment. Additionally, reference lists of key reviews and eligible primary studies were screened to find relevant publications that may not have been identified by the database search.

#### 2.2.1 Search terms and boolean strategy

The key search strategy included a combination of Boolean operators for drug-repurposing terms and AI-related or translational-validation terms. The main search concept was:

(“drug repurposing” OR “drug repositioning” OR “drug reprofiling”) AND (“artificial intelligence” OR “machine learning” OR “deep learning” OR “neural network” OR “knowledge graph” OR “graph neural network*” OR “network medicine” OR “foundation model*” OR “large language model*”) AND (mechanism* OR pharmacology* OR “target engagement” OR pathway* OR biomarker* OR ADMET OR toxic* OR safety OR pharmacovigilance OR “drug-drug interaction*” OR “real-world evidence” OR “electronic health record*” OR “target trial emulation” OR translational OR validation).**

The search syntax was customized to the indexing needs of each database, with database-specific subject headings and field tags used as appropriate. Targeted searches were also carried out for regulatory, reporting and pharmacovigilance guidance related to biomedical decision making with the support of AI. Report of the full database-specific search strings is strongly recommended for reproducibility, Supplementary Table S1.

#### 2.2.2 Eligibility criteria

Researches were eligible when they addressed at least one predefined review domain: (i) AI- or computationally assisted identification of drug-repurposing candidates; (ii) mechanistic or pathway-based interpretation of repurposing predictions; (iii) experimental, causal, or biomarker-based validation; (iv) ADMET, toxicity, contraindication, drug–drug interaction, or pharmacovigilance assessment; (v) external or real-world validation of repurposing candidates; or (vi) methodological, reporting, or regulatory requirements relevant to translational AI. Eligible evidence included peer-reviewed original research, methodological studies, high-quality reviews used to contextualize rapidly developing areas, and authoritative regulatory or reporting guidance. Studies were excluded when they focused exclusively on *de novo* drug discovery without a repurposing component, described AI methodology without relevance to therapeutic candidate evaluation, lacked sufficient methodological or pharmacological information for the review questions, or were not available in English. Preprints were considered only where they addressed rapidly evolving AI methods for which peer-reviewed evidence was limited and were clearly identified as such.

#### 2.2.3 Screening and study selection

Published documents were reviewed for relevance to the established review questions and inclusion criteria. The titles and abstracts were first reviewed and then the full texts were reviewed of the publications that were potentially relevant. Studies with substantive evidence of AI-based candidate generation, mechanistic interpretation, safety assessment, or experimental or real-world validation, and studies on the translation of such studies were given priority. For those publications covering the same model or framework, the report with the most information, and which was published in a peer-reviewed journal, was selected, with complementary publications kept if they included mechanistic, validation, or translational information not provided elsewhere. Two reviewers independently screened and disagreements were solved through discussion and consensus.

### 2.3 Evidence extraction and critical synthesis

An evidence matrix, structured according to the review questions, was created for each included publication. Eligible literature was synthesized based on predefined domains of translation, not just by publication date. These domains encompassed AI methodology and data type, objective of candidate generation, degree of mechanistic interpretability, biological support or causality, experimental validation, dose–exposure feasibility, ADMET and toxicity assessment, drug–drug interaction and contraindication assessment, pharmacovigilance evidence, real-world clinical validation, reproducibility, and regulatory relevance. Particular attention was given to the distinction between computational prediction and pharmacological evidence. Studies were not excluded from the synthesis because of positive or negative findings; contradictory findings, methodological limitations, lack of external validation, and incomplete safety assessment were retained. Positive and negative findings were not a basis for exclusion; contradictory findings, methodological limitations, lack of external validation, and incomplete safety assessment were not a basis for exclusion. This resulted in an evidence map that was then used to highlight the common gaps in translation and to develop the proposed sequenced system. Table 1 presents an evidence map that was developed using the structured extraction to compare representative studies and frameworks with respect to computational approach, mechanistic support, safety assessment, validation strategy and principle translational limitations.

**TABLE 1:** Evidence map of representative studies and frameworks informing AI-driven drug repurposing and translational validation.

Study/approach	AI/analytical approach	Primary repurposing role	Mechanistic evidence	Safety assessment	External/experimental/RWE validation	Key translational limitation
deepDR (Zeng et al., 2019)	Multimodal deep autoencoder	Candidate prioritization	Limited/direct mechanism not central	Limited	Primarily computational	Predictive representation does not establish pharmacological mechanism
Guney et al. (2016)	Network medicine	Network-based drug efficacy prediction	Biological proximity	Not central	Computational	Network proximity does not prove therapeutic direction or dose feasibility
Cheng et al. (2018)	Network medicine + population validation	Candidate prediction	Biological network support	Limited	Population-level and experimental validation	Does not provide a complete mechanism–safety decision pathway
(Himmelstein et al. (2017)	Heterogeneous knowledge graph	Candidate prioritization	Relational paths	Adverse-event relations possible	Limited clinical validation	Graph relation remains associative
KGML-xDTD (Ma et al., 2023)	Knowledge-graph ML	Treatment prediction	Path-based mechanism explanation	Limited	Computational	Explanation does not establish causal efficacy
TxGNN (Huaneg et al., 2024)	Graph foundation model	Indication/contraindication prediction	Multi-hop explanation	Contraindication information	Benchmark/external evaluation	Requires downstream pharmacological and clinical validation
Liu et al. (2021)	Deep learning + trial emulation	Repurposing and clinical validation	Limited relative to mechanism-focused approaches	Clinical data partly informative	Strong RWE validation	Observational causal assumptions remain important
Yan et al. (2024)	Generative AI + EHR analysis	Candidate generation and validation	Incomplete	Not systematically integrated	Two-database RWE validation	Residual confounding and lack of causal confirmation
READUS-PV (Fusaroli et al., 2024)	Pharmacovigilance reporting framework	Safety-signal interpretation	Not applicable	Strong PV focus	Not efficacy validation	Signals cannot establish causality
TRIPOD + AI (Collins et al., 2024)	Reporting guidance	Transparency/reproducibility	Not repurposing-specific	Not specific	Reporting/validation guidance	Does not determine candidate translational readiness
FDA AI guidance (USFa and DA, 2025)	Risk-based regulatory framework	AI credibility assessment	Context-specific	Context-specific	Context-of-use validation	Not a drug-repurposing workflow

## 3 Current AI approaches in drug repurposing: evidence, strengths, and translational limitations

AI drug repurposing is the process of leveraging machine learning models to discover new applications for existing drugs by analyzing the similarities and differences between drug structures and their known therapeutic targets, as well as between the genes, pathways, diseases, phenotypes, and clinical outcomes they affect. Instead of classical machine learning models, which are based on curated features, the field has evolved from deep learning, network medicine, knowledge graphs, graph neural networks, to foundation models. While these strategies have enhanced the accuracy of candidate identification, the importance of these strategies for pharmacology extends beyond the accuracy of the prediction to the potential for contributing to mechanism explanation, safety assessment, and translational validation.

### 3.1 Machine learning-based repurposing

Classical machine-learning methods have been successful in the realm of AI-assisted drug repurposing, with applications ranging from drug–disease association prediction to drug response prediction and candidate prioritization. These approaches include supervised learning methods like logistic regression, support vector machines, random forests, gradient boosting algorithms, and other feature-based models that combine molecular descriptors, chemical fingerprints, drug targets, adverse-effect profiles, gene-expression signatures, and other disease-associated features. Besides classification and ranking, matrix-completion and matrix-factorization are important computational strategies to predict and repurpose drugs. These techniques represent the prediction of missing drug–disease relationships, drug–cell line relationships or drug–response relationships as partially observed matrices and infer missing relationships by learning latent patterns from the available biological and pharmacological data. These methods have been applied to predict therapeutic efficacy, find potential therapeutic drugs and enable precision medicine approaches by correlating molecular features with clinical drug response (Liu et al., 2020). For instance, Xianfang et al. proposed an indicator-regularized non-negative matrix factorization (IRNMF) method for drug repurposing against COVID-19 by incorporating drug-related and disease-associated information to uncover potential therapeutic candidates as a representative example. These methods of matrix factorization prove useful for identifying latent drug–disease connections in the absence of direct therapeutic relationships (Tang et al., 2021).

Classical machine learning is relatively easy to interpret, efficient to compute and has merits in the case of biologically meaningful features. But these models are still very dependent on the completeness and quality of the predefined features. Predicted associations may be based on statistical similarity and not necessarily represent biological mechanism, target engagement, plausibility of dose–response or safety issues specific to individual patients. Thus, classical machine-learning techniques are useful for prioritization at the early stage but need to be tested mechanistically, in experiments, and clinically before their application in translation (Vamathevan et al., 2019; Zeng et al., 2019; Guetari et al., 2023).

### 3.2 Deep learning-based repurposing

Deep learning has enabled models to learn more complex non-linear patterns from high-dimensional biomedical data, ushering in a new era of AI-driven repurposing. Autoencoders, convolutional neural networks (CNNs), recurrent neural networks (RNNs), transformers, attention-based architectures, and multimodal models can process gene-expression profiles, chemical structures, molecular fingerprints, protein sequences, clinical records, images, and features derived from text. For instance, the deepDR framework combined multiple drug-related networks and trained a multimodal deep autoencoder to learn drug representations for *in silico* drug repositioning (Zeng et al., 2019; Al-Zoghby et al., 2025).

Although deep learning models can be very predictive, they can be black boxes. This poses a significant challenge to pharmacological interpretation as the researcher must determine whether the prediction is based on the target biology, the pathway relevance, the pharmacodynamic plausibility, and safety evidence. Moreover, deep learning models can also overfit to biased or incomplete datasets, particularly when positive drug–disease associations are overrepresented relative to negative or unsuccessful associations. The principal translational challenge for deep learning is therefore determining whether its learned representations reflect disease-relevant biology rather than dataset-specific correlations, particularly when model performance is evaluated within the same data ecosystem used for development.

### 3.3 Network medicine and graph-based approaches

Network medicine attempts to ground the concept of drug repurposing in a biologically intuitive way by considering diseases as modules of interacting genes and proteins in the human interactome. In this model, a drug can be considered therapeutically relevant if the targets are near or are shared with disease-associated proteins. Protein–protein interaction networks, drug–target networks, and drug–target–disease networks can then be used to make estimates of biological proximity between drugs and diseases.

Repurposing opportunities have been identified by the quantification of the distance between drug targets and disease proteins, and the use of network proximity. Guney et al., 2016 proposed a network-based approach to *in silico* drug efficacy screening (Guney et al., 2016), and Cheng et al. (2018) integrated network-based prediction with population-level validation and experimental testing (Cheng et al., 2018). These studies demonstrate how network-based approaches can inform biologically grounded candidate prioritization. However, network proximity alone does not establish a therapeutic mechanism. A credible candidate must also show evidence that the relevant target and pathway are modulated in the therapeutically appropriate direction, at a clinically achievable exposure, and within the relevant disease context.

### 3.4 Knowledge graphs and graph neural networks

Biomedical knowledge graphs (BKGs) are particularly valuable for mechanism-aware drug repurposing because they represent drugs, genes, proteins, targets, pathways, diseases, phenotypes, adverse events, biomarkers, and clinical outcomes within a structured relational framework. This structure enables models to infer drug–disease associations while also generating explanatory paths that can support pharmacological interpretation. This is for models to deduce drug–disease associations, as well as explanatory paths that can help with pharmacological interpretation. Hetionet/Rephetio showed that biomedical knowledge can be integrated systematically for prioritizing repurposing candidates across a heterogeneous network (Himmelstein et al., 2017).

Recently, graph neural networks have been introduced that learn complex multi-hop relationships on heterogeneous biomedical graphs. There is also a growing trend in the recent past toward methods that integrate multiple and diverse biological information sources into a single graph, instead of focusing on a single interaction network. To predict drug–target interaction, Yang et al. constructed a drug–target–disease network and introduced a novel framework, called MGCLDTI, which employs multi-view knowledge of drugs, targets and diseases in a heterogeneous graph. The strategy includes global topological representations derived by DeepWalk, graph contrastive learning and node masking to enhance the local structural representations of drugs and targets, and then the drug–target interaction (DDD) is predicted by LightGBM (Yang et al., 2025). This shows the potential of using heterogeneous information fusion and contrastive representation learning to enhance pharmacological predictions by leveraging complementary biological relationships, but predicted drug–target interactions still need to be validated by downstream mechanisms and experiments to be considered evidence for therapeutic efficacy. For instance, Meng et al., developed DRAGNN, a drug–disease neighborhood information augmented GNN (DDN) which integrates graph attention and drug–disease neighborhood information for better drug–disease association prediction, and validated it on three benchmark datasets and then analyzed it through database evidence, molecular docking and drug–disease network analysis, respectively (Meng et al., 2024). For instance, Huang et al. (2023, 2024) developed a graph foundation model called TxGNN to predict indications and contraindications for thousands of diseases by training on a large medical knowledge graph, which is used to guide clinicians during drug repurposing (Huaneg et al., 2024; Huang et al., 2023). Likewise, KGML-xDTD infers drug treatment relationships and provides mechanism-of-action explanations based on knowledge graph paths (Ma et al., 2023), and XG4Repo enables interpretable drug repurposing by graph completion using paths (Jiménez et al., 2024). The comparative advantage of these approaches, therefore, lies in mechanistic traceability rather than causal certainty, with inference quality remaining dependent on graph completeness, relation quality, and the biological validity of the encoded knowledge.

### 3.5 Large language models and foundation models

Large language models and foundation models are newly developed tools for drug repurposing that are well-suited to generate biomedical literature, clinical text, drug labels, guidelines, and structured databases. They can be used to assist literature mining, hypothesis generation, summarizing mechanisms, the development of clinical rationales, and the integration of unstructured knowledge. Structured biomedical knowledge can also be integrated with representation learning to facilitate general therapeutic reasoning, as illustrated by TxGNN’s ranking of potential indications and contraindications for rare diseases (Huaneg et al., 2024).

However, LLMs must be used cautiously in pharmacological research because they may generate fabricated claims, inaccurate citations or quotations, and biologically plausible but unsupported explanations. They may also preferentially reproduce positive findings while omitting negative evidence, dose-related information, or adverse effects. Hence, it is important that LLM candidates and rationales be considered as working hypotheses and not evidence. All outputs should be compared to evidence related to the mechanism of action, toxicity, pharmacovigilance signals, real world evidence, and pharmacology expertise (Kim et al., 2025).

One of the significant advancements in recent times is retrieval-augmented generation (RAG), where an LLM draws on external data sources during generation to augment its parametric knowledge. RAG can provide factual grounding, evidence traceability, and access to the most up-to-date or domain-specific information without needing to store all knowledge in the RAG’s parameters (Lewis et al., 2020). In medicine, the MedRAG framework systematically compared combinations of medical corpora, retrievers, and LLMs, and showed that by augmenting LLM-based question-answering with retrieval, the overall performance could be significantly improved, though the performance is still sensitive to the retrieval strategy and placement of evidence (Xiong et al., 2024). More complex methods like Self-RAG enable the model to obtain evidence dynamically and assess the relevance and support of retrieved evidence (Asai et al., 2024), rather than using static retrieval for every query. To assist in drug repurposing, these methods might enable to support assertions of drug targets, mechanisms, safety, clinical evidence and literature provenance with curated biomedical resources. RAG must be treated as a strategy to mitigate hallucination, however, because irrelevant or incomplete retrieval and incorrect integration of retrieved evidence can still lead to unsupported conclusions, it is important to keep in mind that RAG is not a guarantee of factuality.

Overall, existing AI approaches have substantially increased the capacity to identify candidate drugs for repurposing. Classical machine learning enables efficient ranking, deep learning captures complex biomedical patterns, network medicine provides biological contextualization, knowledge graphs and GNNs facilitate mechanistic reasoning, and foundation models support broad hypothesis generation. A central challenge is the integration of these approaches within a workflow that is focused on validation and is connected to the ability to explain the mechanism, assess safety, and prioritize for clinical relevance. A comparative summary of the major approaches in AI drug repurposing is given in Table 2.

**TABLE 2:** Literature-supported qualitative comparison of major AI methodologies used in drug repurposing.

AI approach	Commonly used data	Reported output/capability	Evidence-supported characteristics	Limitations identified in cited literature	Review-derived translational interpretation	Representative sources
Classical machine learning	Drug and disease features, molecular descriptors, chemical fingerprints, target profiles	Candidate ranking and drug–disease classification	Uses prespecified molecular, drug, target, and disease features to generate candidate rankings or drug–disease classifications; reviewed applications rely on explicitly constructed feature representations	Dependence on prespecified features may restrict representation of complex or latent biological relationships not captured by the selected descriptors	Can support early candidate prioritization when biologically informative variables are available; additional mechanistic and biological validation is required before translational interpretation	(Vamathevan et al., 2019; Guetari et al., 2023; Bi et al., 2025; Hodos et al., 2016)
Deep learning	Omics data, chemical structures, EHRs, images, biomedical text, and heterogeneous biomedical data	Prediction scores, learned representations, embeddings, and candidate prioritization	Learns latent representations from high-dimensional or multimodal biomedical inputs; deepDR provides an example of multimodal representation learning for drug repositioning	Reviewed applications raise concerns regarding overfitting and direct pharmacological interpretability, particularly when learned representations are evaluated within the same or closely related data ecosystem used for model development	Predictive performance should not be interpreted as mechanistic or translational evidence without explanation and independent validation	(Zeng et al., 2019; Al-Zoghby et al., 2025)
Network medicine	Protein–protein interaction networks, disease modules, and drug targets	Network proximity and drug–disease associations	Uses proximity between drug targets and disease-associated molecular modules as a basis for biologically informed candidate prioritization	Network proximity does not independently establish direction of pathway modulation, pharmacodynamic effect, clinically achievable exposure, or therapeutic causality	Useful for biologically informed prioritization, but pathway direction, exposure feasibility, and additional biological evidence remain necessary	(Guney et al., 2016; Cheng et al., 2018)
Knowledge graphs	Drug–target–gene–pathway–disease–phenotype–biomarker–adverse-event relationships	Relational paths and link prediction	Represents heterogeneous biomedical relationships and, in systems such as Hetionet, KGML-xDTD, and path-based graph-completion models, can generate relational paths supporting candidate interpretation	Conclusions depend on the completeness, accuracy, and curation of encoded knowledge; inferred relational paths remain associative and do not independently establish causality	Particularly useful for mechanism mapping, while predicted relationships require independent biological or clinical validation	(Perdomo-Quinteiro and Belmonte-Hernández, 2024; Jiménez et al., 2024; Ma et al., 2023; Himmelstein et al., 2017; MacLean, 2021)
Graph neural networks	Heterogeneous biomedical graphs and multi-relational networks	Drug-repurposing predictions, drug–target interaction prediction, embeddings, and multi-hop graph inference	Processes multi-relational biomedical graph structure for drug–disease prediction; systems such as TxGNN and KGML-xDTD additionally provide multi-hop or path-based explanations	Predicted graph relationships and explanatory paths remain computational inferences and depend on graph quality, training structure, and validation design	Can support relational prediction and mechanism-oriented prioritization, but graph explanations should not be interpreted as causal evidence	((Huaneg et al., 2024; Jiménez et al., 2024; Ma et al., 2023), (Yang et al., 2025), (Meng et al., 2024))
Large language models/foundation models	Biomedical literature, clinical text, drug labels, structured databases, and medical knowledge graphs	Hypothesis generation, rationales, evidence synthesis, and candidate prioritization	Can synthesize information from biomedical text and structured knowledge sources for hypothesis generation and evidence organization; graph foundation models may additionally support indication and contraindication prediction. Retrieval-augmented approaches can additionally ground generation in external biomedical evidence and improve source traceability	Reported concerns include hallucinated or unsupported claims, citation errors, sensitivity to terminology changes, uncertainty in free-form generation, and benchmark contamination. RAG remains dependent on retrieval quality, corpus coverage, and the relevance of retrieved evidence	Best treated as expert-assisted hypothesis-generation and evidence-synthesis tools whose outputs require source verification, uncertainty assessment, and human oversight	(Huaneg et al., 2024; Kim et al., 2025; Lewis et al., 2020; Xiong et al., 2024; Asai et al., 2024; Farquhar et al., 2024; Language Models et al., 2024; North American et al., 2024)

#### 3.5.1 Current limitations of foundation models and large language models

Despite their capacity for large-scale knowledge integration and hypothesis generation, current foundation models and large language models have several limitations that restrict their direct use for translational drug-repurposing decisions. A major concern is hallucination, in which a model generates fluent and apparently plausible statements that are factually incorrect, unsupported, or inconsistent with the underlying evidence. This problem is particularly consequential in pharmacology because a fabricated drug–target interaction, pathway relationship, dose, contraindication, or safety signal could create a mechanistically plausible but erroneous repurposing hypothesis. Importantly, linguistic confidence should not be interpreted as epistemic confidence. Recent work using semantic entropy demonstrated that uncertainty at the level of meaning can identify a subset of LLM confabulations, but also showed that uncertainty estimation does not detect all systematic errors and therefore cannot itself guarantee factual reliability (Farquhar et al., 2024).

Uncertainty estimation remains especially challenging for free-form biomedical generation because several semantically equivalent answers may be expressed using different token sequences, while an incorrect answer may nevertheless be generated consistently. Consequently, conventional token-level probabilities, model self-assessment, or apparent response confidence may provide incomplete measures of uncertainty. Semantic uncertainty, calibrated abstention, retrieval-augmented generation, adaptive retrieval, source-grounded verification, and external evidence checking are potential complementary mitigation strategies that are promising but should be viewed as no silver bullets to prevent hallucination (Farquhar et al., 2024). In drug repurposing, uncertainty should therefore be explicitly characterized when models infer mechanisms, rank candidates, or synthesize evidence, particularly when those outputs are used to determine which candidates progress to experimental or clinical evaluation.

Reproducibility represents a related limitation. Generative outputs can differ due to stochastic sampling and be sensitive to seemingly subtle variations in the representation of biomedical information. The problem is relevant for drug repurposing since the same pharmacological concept can be described by different names (generic, brand, synonym, abbreviation, description, *etc.*). Gallifant et al. showed that the performance of biomedical benchmarks decreased measurably when they replaced the names of generic drugs and brand names (Language Models et al., 2024), indicating that seemingly insignificant linguistic perturbations can impact model performance. For reproducible research, researchers should therefore report the precise model and version, prompt structure, decoding and sampling settings, retrieval sources where applicable, evaluation date, and repeated-run procedures rather than reporting only the final generated output or aggregate performance.

Benchmarking has also proven to be quite difficult. An excellent score on static question-answering tasks does not guarantee a strong ability to perform robust pharmacological reasoning in realistic scenarios. Evaluation examples or very similar material may be in model training data, leading to the appearance of a high-performance rate when in fact the model is contaminated. Deng et al. provided evidence of contamination in a large number of popular benchmarks for LLMs and called for the adoption of better assessment methods (North American et al., 2024). Biomedical robustness studies also demonstrate that performance can vary following clinically and/or pharmacologically small perturbations, suggesting that the generalizability of the benchmarks may be overstated by the models (Language Models et al., 2024). Future assessment of the foundation models used for drug repurposing should thus account for independent and time-separated datasets, perturbation testing, out-of-distribution testing, consistency with the mechanisms, uncertainty calibration and safety-oriented testing, as well as external biological validation, besides the primary focus on benchmark accuracy.

Another restriction is imposed by regulatory translation. Regulatory credibility is related to the context in which the model is applied, the impact of model errors, the representativeness and quality of the input data, model performance, documentation, and the ability to assess and manage model-related risks. The 2025 draft guidance from the U.S. FDA follows a risk-based credibility assessment approach that is directly tied to the context of use of the AI model (USFa and DA, 2025). Likewise, the European Medicines Agency has highlighted “Lifecycle” based aspects of the use of AI throughout the medicinal-product development process, such as data governance, transparency, performance evaluation and for human oversight (Agency, 2024). The regulatory expectations pose specific challenges for foundation models, such as their size of the training data, probability-based results, multi-component architecture and ongoing model refinement which can make them difficult to audit, reproduce, trace and validate. In the meantime, it is advisable to use foundation models as hypotheses generators and evidence synthesizers in a controlled human-in-the-loop workflow and not as sources of regulatory-quality pharmacological evidence.

### 3.6 Critical comparison of AI methodologies for drug repurposing

Classical machine learning, deep learning, network medicine, knowledge graphs, graph neural networks (GNNs), and foundation models differ not only in predictive capability but also in their interpretability, biological grounding, data requirements, and translational relevance. While classical machine-learning techniques are relatively straightforward and efficient, especially when biologically relevant features are known *a priori*, the methods depend heavily on feature selection and may fail to model complex nonlinear relationships in disease biology (Vamathevan et al., 2019; Guetari et al., 2023). Deep-learning models are better able to address some of these shortcomings by learning complex representations from high-dimensional molecular, omics, chemical, imaging, and clinical data, but this increased flexibility comes at a cost of data requirements, risk of overfitting, and loss of pharmacological interpretability (Zeng et al., 2019; Al-Zoghby et al., 2025). Therefore, better prediction is not always associated with better translation credibility if there is no biological basis for the prediction.

Network medicine offers more biological context, by examining relationships between drug targets and molecular modules associated with disease. However, this strategy can also uncover candidates that are biologically close to the disease mechanism, but network proximity does not necessarily imply that the pathway is modulated in the therapeutic direction or at a clinically relevant drug exposure (Guney et al., 2016; Cheng et al., 2018). Designed to enable more explicit mechanistic interpretation, knowledge graphs add to this reasoning by capturing heterogeneous relationships between drugs, genes, proteins, pathways, diseases, phenotypes, biomarkers, and adverse events. However, the validity of their conclusions is dependent on the completeness and quality of the base biomedical knowledge.

GNNs also provide the ability to model complex, multi-hop, multi-relational interactions within biomedical graphs. More recent graph-representation-based methods also incorporate both heterogeneous drug and target and disease data with contrastive learning for better representation quality and drug–target interaction prediction (Yang et al., 2025). Some systems like KGML-xDTD and TxGNN couple pathway or graph-based explanations with candidate prediction which provide more mechanistic traceability than traditional ranking models (Huaneg et al., 2024; Ma et al., 2023). GNNs can however have black-box properties, and an interpretable path in the graph does not imply causality or therapeutic benefit. The most general capability to assimilate biomedical literature, clinical text, structured databases, and heterogeneous knowledge come from foundation models and large language models. The strengths of their main advantages are their general scalability and their ability to synthesize knowledge quickly, but they also come with some drawbacks, such as potentially hallucinating claims, misattributing citations, synthesizing biased knowledge, or delivering knowledge that cannot be explained mechanistically (Kim et al., 2025).

Thus, no single AI methodology is the best for drug repurposing in all cases. While classical machine learning offers more transparency with reduced representational depth, deep learning provides enhanced pattern recognition capabilities at the expense of direct interpretability, network medicine advances biological contextualization capabilities but with a high level of association, knowledge graphs bring better mechanistic traceability, but rely on curated knowledge, GNNs offer better relational prediction but demand careful biological validation and, finally, foundation models offer better knowledge integration but require stringent verification and human oversight. Therefore, methodology selection should be done not only for predictive performance, but also, depending on the intended translational task. A hybrid approach of predictive modelling, graph-based mechanistic reasoning, safety assessment, and independent biological or real-world validation could also provide more robust translatable evidence than any single AI architecture, as shown in Table 2.

Table 2 is not intended to compare the computational efficiency, data needs, prediction abilities, or complexity of the models presented in the reviewed literature, but rather to provide a qualitative synthesis of the methodological characteristics reported. The main distinction between the reviewed approaches is in the nature of the information they process, and the type of output they generate: feature-based machine learning models process information provided as prespecified drug and disease descriptors; deep learning could learn latent representations from high dimensional or multimodal data; network medicine models used the proximity of drugs to disease modules as information; knowledge graph or graph-based models process multi-relational biomedical information and may yield explanatory pathways; foundation models provide support for broader knowledge integration and hypothesis generation. The methodological differences are not sufficient to prove superiority of the translational drug repurposing approach. The relevance to translation, on the other hand, is dependent on the quality of the resulting mechanistic, safety, experimental, and clinical evidence.

Collectively, the literature reviewed thus far indicates that the sophistication of the computations has not been paralleled by the sophistication of the validation that has been done in the translation direction. Classical machine-learning studies typically offer more model transparency but less biological contextualization, deep-learning studies better representation learning but at the cost of interpretability and validation; network or knowledge-graph approaches better contextualize the biological aspects but are mostly associative; and finally, foundation models expand on knowledge integration while new concerns emerge such as hallucination, reproducibility, calibration, and evidence traceability. The most consistent shortcoming is across the methodological families, the lack of consistent evidence of the relationship between predicted candidates and mechanism, exposure feasibility, safety, causal support, and clinically meaningful validation. This is an ongoing trend that serves as the empirical and conceptual foundation for the later presented translational framework.

## 4 Evidence synthesis I: mechanistic and causal validation

Mechanistic awareness is crucial for translating AI-generated drug-repurposing predictions into pharmacologically relevant hypotheses. Computational repurposing involves leveraging AI models to predict potential drug–disease associations that are learned by analyzing the relationships between drugs, targets, genes, proteins, pathways, diseases, phenotypes, adverse events, and clinical outcomes. A predicted association is insufficient unless it can be linked to a biologically plausible mechanism of action within the target disease (Park, 2019). The issue of pharmacological interpretation is not just whether a model predicts a candidate drug, but whether this prediction makes therapeutic sense and is mechanistically plausible, and whether it is safe in the intended clinical setting.

This distinction is important since drug repurposing relies on the existing knowledge in the field of pharmacology. The target, pharmacokinetics, dosage, toxicity, contraindications and adverse effects of repurposed drugs may all be known, but not necessarily applicable to the new indication. A new disease context can mean new tissues, molecular pathways, comorbidities, requirements for exposure, vulnerabilities of patients, background therapies, and so on. In this way, mechanism-aware AI should be able to inform a structured assessment of drug–target interaction, target relevance, pathway modulation, biomarker support, dose–response plausibility, and safety implications prior to the consideration of the candidate being translationally relevant (Wei et al., 2026).

### 4.1 Why mechanism matters in pharmacology

In pharmacology, mechanism of action provides the biological basis for interpreting a drug effect. Mechanism-aware assessment therefore examines whether an AI-prioritized candidate can be connected to a coherent and testable sequence linking drug exposure, target engagement, pathway modulation, biomarker response, and the intended disease phenotype (Milligan et al., 2013). This shifts the emphasis from model confidence to pharmacodynamic plausibility and helps define the evidence required for subsequent translational assessment.

Mechanism-aware interpretation should operate across several levels. At the molecular level, the drug should interact with, inhibit, activate, stabilize, or otherwise modulate a target that is relevant to the disease. The target should be engaged in a biological process associated with the initiation, progression, expression or resistance to a disease at the pathway level. At the pharmacodynamic level, the anticipated effect should correspond to measurable biomarkers or molecular and phenotypic signatures. The drug should have a mechanism of action at clinically achievable concentrations that does not have unacceptable toxicity in the desired population at the translational level (Landry and Gies, 2008).

This is a mechanistic filtering process as AI models can learn correlations on incomplete or biased data. A drug can be predicted on the basis of its similarity to known drugs, its frequent mention in the literature, it having molecular properties similar to those of known drugs, or its targets being in a proximity network to disease associated proteins. These signals can be helpful for prioritization, but could also be a signal of confounding, literature bias, database incompleteness or indirect association. Computational proximity or predicted proximity based on a knowledge graph also have been demonstrated to need biological interpretation before they can be used as a pharmacologic clue (Guney et al., 2016; Cheng et al., 2018; Himmelstein et al., 2017).

### 4.2 Drug–target–pathway–disease reasoning

AI-generated candidates can be assessed for their pharmacological plausibility using drug–target–pathway–disease reasoning. This logic starts with the drug and what is known or predicted to be its targets. It then considers whether these targets are targets of disease-relevant pathways, and whether the modulation of these pathways could be considered to have the potential to change the disease phenotype. AI prediction is thus linked to pharmacodynamics, therapeutic plausibility and experimental testability.

For instance, the re-purposed drug could be an inhibitor of a pro-inflammatory enzyme, block an immune receptor, affect a pathway of kinases associated with cancer progression, or affect a transporter associated with metabolic disease. Whether the prediction is on a tissue specific basis, if the pathway is active under disease conditions, whether modulation is in a therapeutic direction, and whether the exposure required to modulate the pathway is clinically achievable are all important factors. Mechanism-aware repurposing should not only be based on the assertion that a drug is “associated” with a disease, but should also involve a biological pathway through which the drug might affect disease biology (Usha et al., 2021).

It should also consider indirect pathways, pathway compensation, off-target effects and polypharmacology. Some repurposed drugs might not directly target the primary disease driver, but could affect disease microenvironments, metabolic pathways, immunity or downstream effectors, and upstream regulators. Polypharmacology may be useful in the case of complex diseases where several targets can interact with each other in different pathways. But sometimes the same multi-target activity is responsible for greater adverse effects, drug–drug interactions, or disruption of pathways (Anighoro et al., 2014). Therefore, both therapeutic and safety implications should be considered when assessing mechanism-awareness.

Network medicine is an important foundation for this sort of reasoning. In network-based repurposing, the proteins associated with a disease are often considered as modules in the human interactome and the candidates for drug development are prioritized by their proximity to the disease modules. Guney et al., 2016 demonstrated that therapeutic drug targets tend to cluster around disease-associated proteins (Guney et al., 2016) and Cheng et al. (2018) further advocated the idea by integrating network prediction and population screening (Cheng et al., 2018). These studies are useful for biologically informed prioritization, but do not demonstrate a therapeutic mechanism when using network proximity. Functional pathway relevance, direction of modulation, dose feasibility, tissue context, and safety evidence are still required for a translational interpretation.

### 4.3 Explainable AI and knowledge graph paths

Explainable AI plays a crucial role in mechanism-aware drug repurposing, where pharmacologists, clinicians, toxicologists, and reviewers should understand the rationale behind the prediction of a candidate. Technical outputs like feature importance, embeddings, attention weights, or model confidence are not the only types of explanations that should be considered. A clinically meaningful explanation should clarify how the candidate drug interacts with relevant targets and how those interactions relate to pathways, biomarkers, disease phenotypes, and potential safety signals.

One of the most important features of biomedical knowledge graphs is the ability to connect drugs, genes, proteins, pathways, diseases, phenotypes, biomarkers and adverse events. Relationships can include binds, inhibits, activates, regulates, treats, causes, increases risk of and contraindicated in. The structure enables AI systems to not only predict links between a candidate drug and a target disease but also to provide interpretable connections. A recent review highlights the value of knowledge graphs for drug repurposing, as they are able to combine heterogeneous biomedical data and facilitate mechanism-oriented inference (Perdomo-Quinteiro and Belmonte-Hernández, 2024; MacLean, 2021).

The pathway can be a sequence of drug, target, pathway, biomarker and disease phenotype. This type of explanation is more useful than a score prediction since it facilitates the appreciation of whether each step is biologically feasible. A more credible candidate will be one for which the drug has an action against a disease-relevant target, the target is involved in a disease pathway, the pathway can be measured by biomarkers, and the drug is expected to impact the disease phenotype.

There are several knowledge graph-based approaches that have tried to mitigate the limitations of the black-box approach in drug repurposing. Del Valle et al. (2018) created Hetionet/Rephetio by connecting biomedical information between drugs, diseases, genes, pathways, symptoms and side effects (del Valle et al., 2018). Ma et al. (2023) developed KGML-xDTD for predicting drug–disease treatment probabilities and for knowledge graph path-based mechanism descriptions (Ma et al., 2023). Jiménez et al. (2024) proposed a path-based explainable knowledge graph completion model for drug repurposing (Jiménez et al., 2024), and Huang et al. (2024) proposed a foundation model trained on a large medical knowledge graph (KGs) for therapeutic indication and contraindication prediction, which can generate explanatory paths with multi-hop (Huaneg et al., 2024).

The main difference is between the technical and pharmacological explainability. A technical explanation might present how a model made a prediction, while a pharmacological explanation should provide an understanding of the mechanism of action of the drug to a biological target, the mechanism of action of the target to the biology of the disease, the direction of the effect (assumed therapeutic or otherwise) and whether the same mechanism could lead to safety issues. The concept structure of mechanism-aware biomedical knowledge graph reasoning is shown in Figure 1.

**FIGURE 1:**
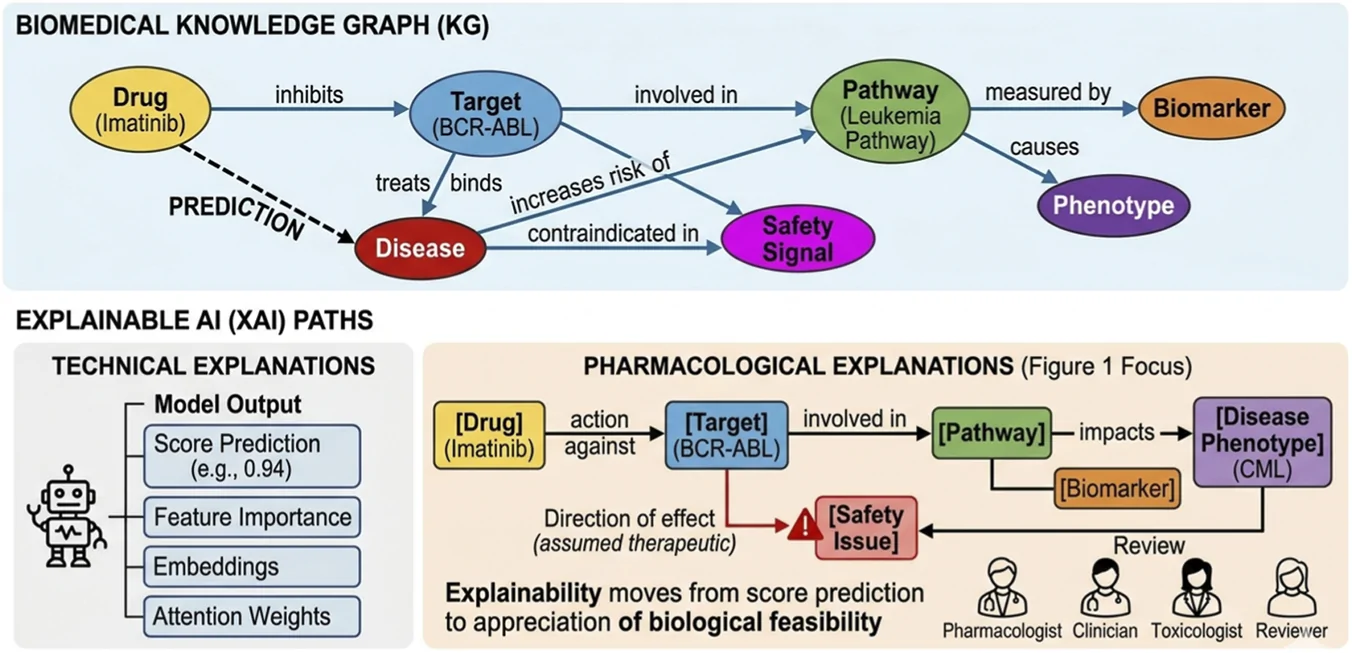
Mechanism-aware biomedical knowledge graph for drug repurposing.

Figure 1 should not be regarded as evidence that a predicted pathway of the graph is a valid mechanism. While the biological validity of any drug–target–pathway–biomarker–phenotype link is still tied to how complete and accurate the knowledge graph underlying the link is, the direction and tissue specificity of the relationships encoded in the graph, achievable drug exposure in the clinic and independent experimental or clinical validation. Furthermore, graph paths can exclude compensating pathways, off-target effects, polypharmacology, conflicting pathways and safety concerns from the source data. Therefore, the primary goal of the figure is to identify a technical model explainability from a pharmacologically testable model reasoning, and further steps should be accompanied by the causal and biological evidence described in Section 3.4.

### 4.4 Causal biological evidence

Mechanism-aware explanations can improve interpretability but do not, by themselves, establish causality. The associations learned by AI models can be based on biomedical databases, literature, molecular profiles, networks, or clinical records, but these associations can be a reflection of confounding, biased data availability, publication trends, or resemblance to already known therapy (Chen et al., 2025). Hence, a higher level of biological and causal evidence is required for successful support of AI predictions in translational pharmacology.

Complementary evidence sources can be helpful to provide causal support. Perturbation data can indicate if the drug influences gene expression, protein activity, pathway activity or cellular phenotype in a way that is consistent with its proposed therapeutic effect. Causal target relevance can be reinforced with genetic evidence using Mendelian randomization (MR). MR employs genetic variations as ‘instrumental variables’ to determine if a genetic perturbation of a biological exposure or drug target is causally associated with disease outcomes. In drug-target MR, cis-acting variants in or near the target gene can be used to approximate target perturbation, which can be used to support or refute the therapeutic rationale for a drug-target MR. But at the same time, these should be carefully considered when interpreting: instrument strength, linkage disequilibrium, horizontal pleiotropy and tissue-specific effects (Schmidt et al., 2020). Pharmacodynamic Plausibility can be supported by evidence of biomarkers if the drug affects measurable markers that can be associated with disease activity, treatment response, or clinical phenotype (Karczewski et al., 2013).

Real-world evidence can also aid a causal interpretation when analyzed using the proper methodology. EHR, claims databases, and observational cohorts can assess if exposure to the putatively effective drug is linked to better outcomes or decreased risk of disease. But there are issues of confounding by indication, treatment selection bias, missing data, exposure misclassification and differences in baseline risks that must be dealt with in these analyses. Some of these limitations can be mitigated by emulating a randomized trial in the available data or by using causal inference techniques. This method was shown by Liu et al. (2021), who used deep learning and actual patient information to simulate clinical trials to test candidates for repurposing (Liu et al., 2021).

Table 3 summarizes the principal evidence domains used in this review to assess mechanistic and causal support for AI-generated repurposing candidates. Target relevance, pathway plausibility, biomarker/pharmacodynamic support, safety assessment and experimental or clinical evidence are all required for greater translational confidence, in addition to computational association supporting hypothesis generation.

**TABLE 3:** Review-derived domains of pharmacological evidence for assessing AI-driven drug-repurposing candidates.

Evidence domain	Key evidence question	Suitable evidence sources	Contribution to translational assessment	Representative supporting literature
Computational association	Does the AI model identify a plausible drug–disease relationship?	Drug–disease databases, similarity measures, machine-learning outputs, matrix factorization, graph link prediction	Provides an initial hypothesis for subsequent pharmacological evaluation; does not itself establish therapeutic efficacy	(Vamathevan et al., 2019; Zeng et al., 2019; Guney et al., 2016; Cheng et al., 2018; Himmelstein et al., 2017; Hodos et al., 2016)
Target relevance	Does the drug interact with a target implicated in the disease?	DrugBank, ChEMBL, BindingDB, target databases, genetic association, cis-Mendelian randomization, and target-expression evidence	Connects predicted drug action to disease-related molecular biology	(Guney et al., 2016; Cheng et al., 2018; Himmelstein et al., 2017; Park, 2019; Wei et al., 2026; Milligan et al., 2013; Landry and Gies, 2008)
Pathway plausibility	Is the target involved in a disease-relevant pathway, and is the expected direction of modulation pharmacologically plausible?	KEGG, Reactome, Gene Ontology, pathway enrichment, disease-module and PPI analyses	Supports interpretation of how target modulation could influence disease biology	(Guney et al., 2016; Cheng et al., 2018; Himmelstein et al., 2017; MacLean, 2021; Park, 2019; Usha et al., 2021; Anighoro et al., 2014; del Valle et al., 2018)
Biomarker/pharmacodynamic support	Does drug exposure alter measurable molecular or phenotypic markers relevant to the proposed mechanism?	Gene-expression signatures, perturbation datasets, LINCS/CMap, proteomics, metabolomics and biomarker studies	Provides evidence that predicted target/pathway modulation produces measurable biological effects	(Milligan et al., 2013; Karczewski et al., 2013; Thombre et al., 2026)
Safety and toxicity	Is the candidate pharmacologically and clinically acceptable in the proposed indication, dose and population?	ADMET models, toxicology databases, labels, contraindications, DDI resources and pharmacovigilance data	Identifies indication-specific toxicity, interaction and population-level safety concerns	(Pu et al., 2019; Fusaroli et al., 2024; Food, 2022; Lee et al., 2025; Vogel et al., 2020; Zhang et al., 2025)
Experimental/clinical support	Is the computational hypothesis supported by experimental, real-world or clinical observations?	Biochemical assays, cell and organoid studies, animal models, EHRs, claims data, observational cohorts, trial emulation and clinical studies	Tests whether the predicted relationship is observable beyond the originating computational model and informs progression to further evaluation	(Liu et al., 2021; Yan et al., 2024; Thombre et al., 2026; Agamah et al., 2020; Hernán and Robins, 2016)

## 5 Evidence synthesis II: safety, toxicology, and pharmacovigilance

This section examines the indication-specific safety evidence required for AI-prioritized repurposing candidates, including ADMET, toxicity, drug–drug interactions, contraindications, and pharmacovigilance.

### 5.1 Why safety must be reassessed in repurposing

Safety does not apply to a specific drug in all situations. A drug that is safe in one indication may have different risks in older adults, pediatric patients, pregnant patients, with hepatic and renal impairment and/or multiple concomitant medicines. In particular, this applies when the AI system is used for repurposing, as many models focus on choosing candidates based on efficacy-related features like similarity to a drug, proximity to pathways, gene-expression reversal, or prediction using graphs, rather than toxicity, contraindications, dose feasibility, or patient-specific risk. While machine learning output needs to be interpreted in a biological, clinical and safety context, the safety assessment needs to be considered early in candidate prioritization (Vamathevan et al., 2019).

### 5.2 ADMET and toxicity prediction

Absorption, distribution, metabolism, excretion, and toxicity are assessed in ADMET prediction, and it can be beneficial in determining pharmacokinetic or toxicological restrictions prior to experimental or clinical validation of a candidate. For AI-driven repurposing, molecular descriptors, chemical fingerprints, structural alerts, toxicogenomic signatures, assay data, graph-based molecular representations, and others can be used to estimate risks like hepatotoxicity, cardiotoxicity, nephrotoxicity, neurotoxicity, reproductive toxicity, genotoxicity, immunotoxicity, and mitochondrial toxicity (Lee et al., 2025; Zhang et al., 2025). Computational prediction of ADMET properties, however, should be approached with caution since it may not fully reflect effects of chronic treatment, immune-mediated toxicity, or disease-related pharmacokinetics changes or population-specific vulnerability of active metabolites or subpopulations. Therefore, ADMET screening is useful in the early stages of risk identification, but is not a substitute for experimental toxicology, clinical pharmacology or safety assessment in real patients.

### 5.3 Drug–drug interaction and contraindication risks

Drug–drug interaction screening is critical when repurposed drugs are suggested to clinical populations currently taking standard drugs. They can interact with each other through cytochrome P450 enzymes, drug transporters, protein binding, additive pharmacodynamic effects, prolongation of the QT interval, organ toxicity and immune-mediated adverse effects. FDA guidance focuses on the consideration of drug interactions involving CYP enzymes and transporters and the communication of clinically relevant drug interaction risks to drug labels (FDA, 2020). Assessment of contraindications is also relevant since drugs with hepatotoxic, nephrotoxic, cardiotoxic and/or QT-prolonging properties might not be appropriate for certain disease groups or co-medications. Hence, it is important to include DDI screening, contraindication review, dose feasibility and population-specific safety assessment before applying AI-based repurposing for translation.

### 5.4 Pharmacovigilance and adverse-event signal evaluation

Pharmacovigilance data are essential for post-marketing evidence in drug safety evaluation for drug repurposing. Spontaneous reporting systems like FAERS, VigiBase, and EudraVigilance are able to detect known and unexpected adverse-event patterns, uncommon toxicities, drug–event associations, and safety concerns that might not be adequately detected in pre-approval trials (Vogel et al., 2020). These systems, however, are subject to underreporting, stimulated reporting, reporting bias, duplicate reporting, missing clinical data, confounding by indication and co-medication effects, and lack of denominator data. As such, disproportionality indicators do not prove a causal relationship. Individual case safety reports (ICSRs) should be carefully reported and interpreted within the context of disproportionality analysis (Fusaroli et al., 2024). Pharmacovigilance should thus be seen as supporting the overall safety assessment, and not as an independent assessment of safety or harm in AI-driven repurposing.

A more recent disease- and drug-specific example is provided by Liu et al., who assessed post-marketing safety of the FcRn antagonists efgartigimod alfa and rozanolixizumab for the treatment of myasthenia gravis using the FAERS database. The study employed reporting odds ratio (ROR) and proportional reporting ratio (PRR) disproportionality analyses to uncover both commonly reported adverse events and potential signals of safety not previously identified. Atrial fibrillation and peripheral neuropathy were among the signals for efgartigimod alfa, and meningitis and hypersomnia were among the signals for rozanolixizumab (Liu et al., 2025). This example demonstrates the utility of spontaneous reporting data for detecting and monitoring possible drug-specific safety signals that might require additional studies and clinical surveillance. Importantly, such disproportionality signals suggest statistical associations, but not necessarily causality, and thus need to be confirmed with complementary clinical or epidemiological evidence.

### 5.5 Safety validation gap in AI repurposing studies

Across the reviewed literature, indication-specific safety evidence remains less consistently integrated than efficacy-oriented candidate prediction, particularly for toxicity, dose feasibility, drug–drug interactions, contraindications, and post-marketing safety signals. Table 4 summarizes these recurring safety-validation gaps and their translational implications.

**TABLE 4:** Literature-supported safety domains relevant to AI-driven drug repurposing.

Safety domain	Evidence/data sources	Principal purpose	Evidence limitation	Representative supporting literature
ADMET assessment	Chemical, pharmacokinetic and molecular-property databases	Identify potential absorption, distribution, metabolism, excretion and toxicity liabilities before further development	Computational estimates may not capture chronic exposure, active metabolites, disease-specific pharmacokinetics or patient-level vulnerability	(Lee et al., 2025); (Zhang et al., 2025)
Toxicity assessment	In silico toxicology platforms, toxicogenomic datasets and assay databases	Identify organ-specific and molecular toxicity signals	Computational findings require experimental or clinical confirmation	(Lee et al., 2025); (Zhang et al., 2025)
Pharmacovigilance	FAERS, VigiBase, EudraVigilance and individual case safety reports	Detect known or emerging post-marketing adverse-event signals	Underreporting, reporting bias, missing denominators, duplicate reports and confounding limit causal interpretation	(Fusaroli et al., 2024); (Vogel et al., 2020); (Liu et al., 2025)
Drug–drug interaction and contraindication assessment	Regulatory labels, interaction databases, CYP/transporter information and pharmacological data	Evaluate polypharmacy, exposure-related interactions and contraindications in the proposed population	Interaction risk varies according to dose, co-medication, population and clinical context	(FDA, 2020)
Real-world safety evidence	EHRs, claims databases, registries and observational cohorts	Assess adverse outcomes and safety patterns in clinically relevant populations	Confounding, missing data, exposure/outcome misclassification and variable data quality affect interpretation	(Liu et al., 2021); (Yan et al., 2024); (Hernán and Robins, 2016)
Expert pharmacology review	Pharmacologists, toxicologists, clinicians and regulatory specialists	Integrate mechanism, exposure, toxicity, interaction and patient-context information	Expert judgement may vary unless assessment criteria and decision rules are defined prospectively	(Collins et al., 2024); (DiFrancesco, 2026)

## 6 Evidence synthesis III: experimental, real-world, and regulatory validation gaps

### 6.1 Computational prediction versus clinical use

Computational performance and translational utility represent different levels of evidence. Metrics such as ranking accuracy, AUC, precision, recall, or link-prediction performance characterize model behaviour but do not directly determine whether a candidate is pharmacologically suitable for a new indication. Translational assessment additionally depends on mechanism, achievable exposure, safety, generalizability, and clinically relevant evidence (Pu et al., 2019) (Vamathevan et al., 2019). The following subsections therefore examine the distinct external, experimental, real-world, reproducibility, and regulatory gaps that influence progression from computational prioritization to clinical development.

### 6.2 External validation problem

A crucial step in the process of determining whether an AI model can generalize beyond the set of data it was trained on is external validation. Most of the re-purposing studies use internal test sets constructed from the same databases, curation logic, or disease–drug knowledge bases on which they are trained. This design may overestimate performance because models can exploit dataset-specific patterns, duplicated knowledge, or latent similarities rather than learning generalizable drug–disease relationships.

Independent validation is needed to confirm predictions across various data sets, disease regions, biomedical knowledge sources, and patient populations. This is especially crucial as drug repurposing databases can be incomplete and skewed toward drugs that are generally more studied, targets, diseases, and positive results. Transparent reporting of data sources, model development and validation strategy, model limitations and generalizability to prediction models is directly applicable to AI-driven repurposing (Collins et al., 2024). In addition to performance metrics, however, external validation should be based on predicted candidate targets that are also relevant to the target, plausible to the pathway, safe, and supported by independent biological or clinical evidence.

### 6.3 Experimental validation problem

One of the main shortcomings of many AI-driven drug repurposing studies is the lack of experimental validation. Computational modelling frequently identifies candidate molecules that are not tested to see if the candidate drug has an effect on the proposed target, pathway, biomarker, or disease phenotype (Agamah et al., 2020). If the effect is not biochemical, cell-based, organoid, animal, target-engagement, or mechanistic, it is difficult to determine if the effect is real, dose relevant and biologically meaningful.

Experimental validation must be carried out to test the predicted mechanism and not just for general biological activity. For example, when a model predicts pathway inhibition, experimental studies should determine whether the candidate modulates that pathway at clinically relevant concentrations. When prediction is based on a reversal of the gene expression pattern, experiments to validate should include testing for the production of the predicted molecular signature and testing for the association of this signature with disease-relevant phenotypes. Mechanism-aligned experiments can therefore determine whether the computationally proposed relationship is observable at pharmacologically relevant exposure and whether target engagement or pathway modulation produces the anticipated biomarker or disease-relevant phenotypic response (Thombre et al., 2026). A helpful example of this is the use of a peptide-based molecular system of Lu et al., where the Pb^2+^ recognition resulted in an experimentally measurable sensing response and facilitated molecular logic operations (Lu et al., 2021). Selective detection of the presence of Pb^2+^ was shown with a detection limit of 0.75 nM, the report of which illustrates how a defined molecular interaction can be transformed to a measurable, testable functional output. This study was not undertaken to examine the potential for AI to drive a “repurpose” process; it nonetheless represents the general methodological principle that computational or conceptual molecular relationships become more compelling when they can be tied to an experimentally demonstrated molecular recognition, response or functional readout. For drug repurposing, the analogous requirement is to determine if a predicted drug–target or drug–pathway relationship leads to the desired target-engagement, biomarker, pathway or phenotypic effect under pharmacologically relevant conditions.

Just as crucial to assessing the translational potential of AI-powered repurposing are unsuccessful experiments. Equally as critical as successful trials are the failures that occur in the experiments. For instance, Johnston et al. employed the NLP screening of ∼1.3 million MEDLINE abstracts to prioritize a list of 3,539 already available drugs for testing in l-DOPA induced dyskinesia, and chose lorcaserin, acamprosate and ganaxolone for preclinical testing. Even though acamprosate and ganaxolone are administered at high doses, they did not show efficacy in the rodent model. However, Lorcaserin was active in antidyskinetic assays and was tested in dyskinetic macaques, where treatment caused a marked increase in parkinsonian disability, so the authors did not support the repurposing of any of the three candidates for this indication. This example illustrates that any highly ranked, AI-driven candidate does not always translate to experimental efficacy or an acceptable benefit–risk profile, and negative validation should not be avoided in the repurposing pipeline (Johnston et al., 2024).

Furthermore, the failure to predict candidates should be reported as a part of the AI-repurposing studies to help define the limitations of the model and to avoid selectively validating only the successful candidates, as the failure is also evidence of the model.

### 6.4 Real-world evidence and trial emulation

Real world evidence is useful in closing the loop between computational prediction and clinical relevance through the use of electronic health records, claims databases, registries and observational cohorts for the assessment of drug exposure, patient characteristics, outcomes, comorbidities, concomitant medicines and safety events. Drug repurposing can be used to determine that patients who have been given a candidate drug exhibit an outcome pattern that matches the one that was predicted to occur if the drug acted as its intended therapeutic target, especially if clinical trials were not yet possible.

Interpretation of the observational real-world analyses should be considered with caution due to immortal-time bias, confounding by indication, treatment-selection bias, differences in baseline risk and exposure and outcome misclassification. The target trial framework put forth by Hernán and Robins offers a structure to minimize avoidable design biases by starting with a description of the randomized trial that would ideally answer the causal question and then mimicking this protocol with observational data. Eligibility criteria, treatment strategies, treatment-assignment procedures, the timing and duration of follow-up, outcomes, causal contrasts, and the plan for statistical analysis should be clearly stated in the target trial (Hernán and Robins, 2016). In particular, eligibility, treatment assignment, and the beginning of follow-up should be tied to a common time zero to minimize immortal-time and selection biases. The causal contrast should also be prespecified, such as it may be an approximation of intention to treat versus per-protocol effect. Emulation of the targeted trial design is therefore a study design specification–before–rather than a process of using an outcome prediction algorithm on observational data.

This framework is important for AI-driven drug repurposing after the candidate generation as a principled way to determine if exposure to an AI prioritized drug is associated with clinically relevant outcomes within a specified causal design. One such application is described by Liu et al. (2021) where deep learning and real patient data was applied to simulate clinical trials for repurposing candidates (Liu et al., 2021). Target trial emulation, however, is best thought of as a causal-design methodology that may include suitable active comparators, adjustment for baseline confounder(s), sensitivity analyses, and a well-defined treatment strategy and estimand. It can enhance the real-world validation of AI-generated candidates, but it has some limitations and residual and unmeasured confounding are important, and observational emulation should not be treated as a randomized trial.

These examples demonstrate that real-world evidence can be used to enhance the translational plausibility, provided that there are appropriate controls for bias, exposure definition, outcome validity, and confounding.

### 6.5 Regulatory and reproducibility gap

Translational value of predictions for AI-based repurposing requires transparency, reproducibility, auditability and clinical interpretability. To ensure the reproducibility of the results, the data source, inclusion and exclusion criteria, data preprocessing, feature construction, model architecture, training strategy, validation design, evaluation metrics, uncertainty analysis, and limitations should be clearly reported.

The adoption of AI-generated evidence, rather than the use of AI, is driven by the trustworthiness of the AI model and the output it generates for a clearly defined regulatory context of use. In the proposed guidance for 2025, the FDA has outlined a risk-based framework for determining how credible the AI model is, which would require sponsors to define the regulatory question of interest, consider the potential consequences of an inaccurate model output, and create a plan for assessing the credibility of the AI model that is commensurate with the risk. These include clear and transparent documentation of how the model was developed and trained, fit-for-use and representative data, prespecified model-performance and acceptance criteria, independent evaluation if appropriate, documentation of deviations, and an explicit determination as to whether the model has sufficient performance for the intended context of use (USFa and DA, 2025).

In the context of AI-driven drug repurposing, this distinction is crucial since a computational candidate generation cannot be viewed as proof of concept for the regulatory pathway. While the FDA guidance does not apply to AI tools that are only applied to drug discovery, if AI-derived analysis is used to inform regulatory decisions on safety, effectiveness, and/or quality, the credibility of the analysis may come into play during regulatory review. This would mean that regulatory readiness would need to have traceable data and model provenance, repeatable analyses, management of bias and uncertainty, clinically relevant validation, documentation of human oversight, and lifecycle processes that would identify changes in performance or data drift. The EMA also stresses on a human-centric, lifecycle-based approach that takes into account data governance, transparency, technical performance, regulatory compliance, and human oversight, and a scientific or regulatory interaction is encouraged at an early stage if an AI system may impact the benefit–risk assessment of a medicinal product (EMA, 2024).

Consequently, the purpose of the evidence should be commensurate with the regulatory impact of AI. A repurposing hypothesis generated by an AI system could be held to a much lower standard of regulatory oversight than a model whose output directly influences patient selection, efficacy evaluation, safety evaluation, dose selection or another decision required for patient safety or efficacy. The higher the regulatory impact of model error, the more robust are the validation, documentation, risk reduction, and regulatory interaction. Therefore, early communication with regulatory agencies would be recommended if AI-generated evidence is going to be used to support clinical development or a marketing application. The key challenges and recommended solutions for the validation of AI-based drug repurposing are summarized in Table 5.

**TABLE 5:** Literature-supported translational validation gaps and review-derived recommendations for AI-driven drug repurposing.

Validation domain	Evidence-supported recurring gap	Representative supporting literature	Translational implication	Review-derived recommendation
Mechanism	Candidate predictions may be generated without direct target-, pathway- or pharmacodynamic validation	(Perdomo-Quinteiro and Belmonte-Hernández, 2024), (Jiménez et al., 2024), (Ma et al., 2023), (Guney et al., 2016; Cheng et al., 2018; Himmelstein et al., 2017), (Huang et al., 2023), (MacLean, 2021; Park, 2019; Wei et al., 2026; Milligan et al., 2013; Landry and Gies, 2008; Usha et al., 2021; Anighoro et al., 2014; del Valle et al., 2018; Chen et al., 2025)	The predicted drug–disease relationship may remain biologically associative rather than mechanistically supported	Evaluate drug–target–pathway–biomarker/phenotype relationships and the expected direction of modulation
Safety	Efficacy-oriented candidate prioritization may not incorporate indication-specific toxicity, contraindications, DDI or post-marketing safety evidence	(Fusaroli et al., 2024); (Lee et al., 2025); (Vogel et al., 2020); (FDA, 2020)	Prior safety in another indication may not establish safety at a new dose, duration, route or population	Integrate ADMET, organ-specific toxicity, dose feasibility, DDI, contraindication and pharmacovigilance assessment before advancement
External validation	Evaluation within the same data ecosystem can permit dataset-specific patterns or duplicated knowledge to influence performance	(Collins et al., 2024)	Reported model performance may not generalize to independent datasets, knowledge sources or patient populations	Use independent datasets, temporal or cross-database validation, and appropriate sensitivity analyses
Experimental validation	Some computational candidates are not subjected to mechanism-aligned laboratory or biological testing	(Agamah et al., 2020)	It remains uncertain whether predicted target/pathway effects occur at pharmacologically relevant exposures	Use biochemical, cellular, organoid, animal, target-engagement or biomarker assays aligned with the predicted mechanism
Explainability	Model explanations may remain technical—such as scores, embeddings or attention—without demonstrating pharmacologically interpretable mechanism	((Perdomo-Quinteiro and Belmonte-Hernández, 2024), (Jiménez et al., 2024); (Ma et al., 2023); (Huang et al., 2023); (MacLean, 2021), (Jain and Wallace, 2019); (Schenone et al., 2013))	Technical interpretability alone does not establish causal or therapeutic plausibility	Translate model explanations into explicit drug→target→pathway→phenotype reasoning and evaluate associated safety implications
Real-world validation	Observational validation may be absent or vulnerable to confounding, treatment-selection bias and exposure/outcome misclassification	((Liu et al., 2021), (Yan et al., 2024), (Hernán and Robins, 2016))	Clinical associations may be difficult to interpret causally	Apply appropriate comparators, exposure/outcome definitions, sensitivity analyses and, where appropriate, target-trial emulation
Reproducibility	Insufficient reporting of data, preprocessing, model settings, validation design or uncertainty can restrict independent verification	(Collins et al., 2024), (USFa and DA, 2025), (Farquhar et al., 2024; Language Models et al., 2024; North American et al., 2024; Agency, 2024), (DiFrancesco, 2026)	Results may be difficult to reproduce, audit or evaluate for regulatory use	Report model/data provenance, preprocessing, model/version settings, validation strategy, uncertainty and limitations transparently
Regulatory readiness	AI outputs may be reported without a defined context of use, risk assessment, credibility plan, or lifecycle controls	(USFa and DA, 2025)	Computational evidence may be insufficiently documented or validated for use in regulatory decision-making	Define the regulatory question and context of use, assess model risk, use fit-for-use data, prespecify performance criteria, document validation and uncertainty, maintain human oversight, and plan lifecycle monitoring and early regulatory engagement

## 7 Proposed translational pharmacology implementation framework

The previous review highlights the fragmentation of evidence across all the processes of computational prediction, mechanistic interpretation, causal assessment, safety evaluation, pharmacovigilance, experimental testing, real world validation, and regulatory assessment. The proposed framework is thus presented as an amalgamation of these gaps and evidence needs from the literature. It is intended to be used to structure the results of the review into a linear decision-making process that can be followed for candidates to be repurposed for AI.

The proposed framework for translational pharmacology was proposed by examining the scope and decision logic of representative computational, mechanistic, real-world, reporting, pharmacovigilance, and regulatory approaches that are relevant to the process of AI-assisted drug repurposing. Existing methods focus on different aspects of the translational pathway. Most of the network and knowledge-graph-based methods focus on the enhancement of the candidate prediction, biological contextualization, and mechanistic interpretability (Huaneg et al., 2024; Jiménez et al., 2024; Ma et al., 2023; Guney et al., 2016; Cheng et al., 2018; Himmelstein et al., 2017), while real-world repurposing methods rely on observational patient data or emulated trials to test clinical associations following candidate generation (Liu et al., 2021; Yan et al., 2024). Transparent reporting and reproducibility of prediction models is the centerpiece of TRIPOD + AI (Collins et al., 2024), the FDA AI credibility framework assesses prediction model reliability based on context of use and regulatory risk (USFa and DA, 2025); and READUS-PV outlines standards for reporting pharmacovigilance signals (Fusaroli et al., 2024). These methods are therefore complementary, but they are implemented at different steps, and do not constitute a single candidate-level pathway from computational prediction, via mechanism, causal plausibility, indication specific safety, pharmacovigilance, real world validation, multidisciplinary review to a clear next translational action.

Figure 2 presents an idealized sequence to explain the proposed framework, but it is not meant to be interpreted as linear. Evidence collected from any subsequent phase may necessitate a re-evaluation of evidence collected in an earlier phase, for instance, an adverse pharmacovigilance signal might lead to a re-evaluation of earlier evidence, or conflicting experimental and real-world evidence might lead to a re-evaluation of the original causal hypothesis. Likewise, the quantity and sequence of evidence that will be available will depend on the drug, indication, available clinical experience, and proposed next steps for the translation. While favorable evidence elsewhere may support a progression, the safety concerns can stop the progression, and missing or conflicting evidence can lead to further validation instead of the progression. The figure is, therefore, not a validated universal pathway of development but rather an organizing decision architecture, whose gates and prioritization rules need to be evaluated in prospective settings of re-purposing.

**FIGURE 2:**
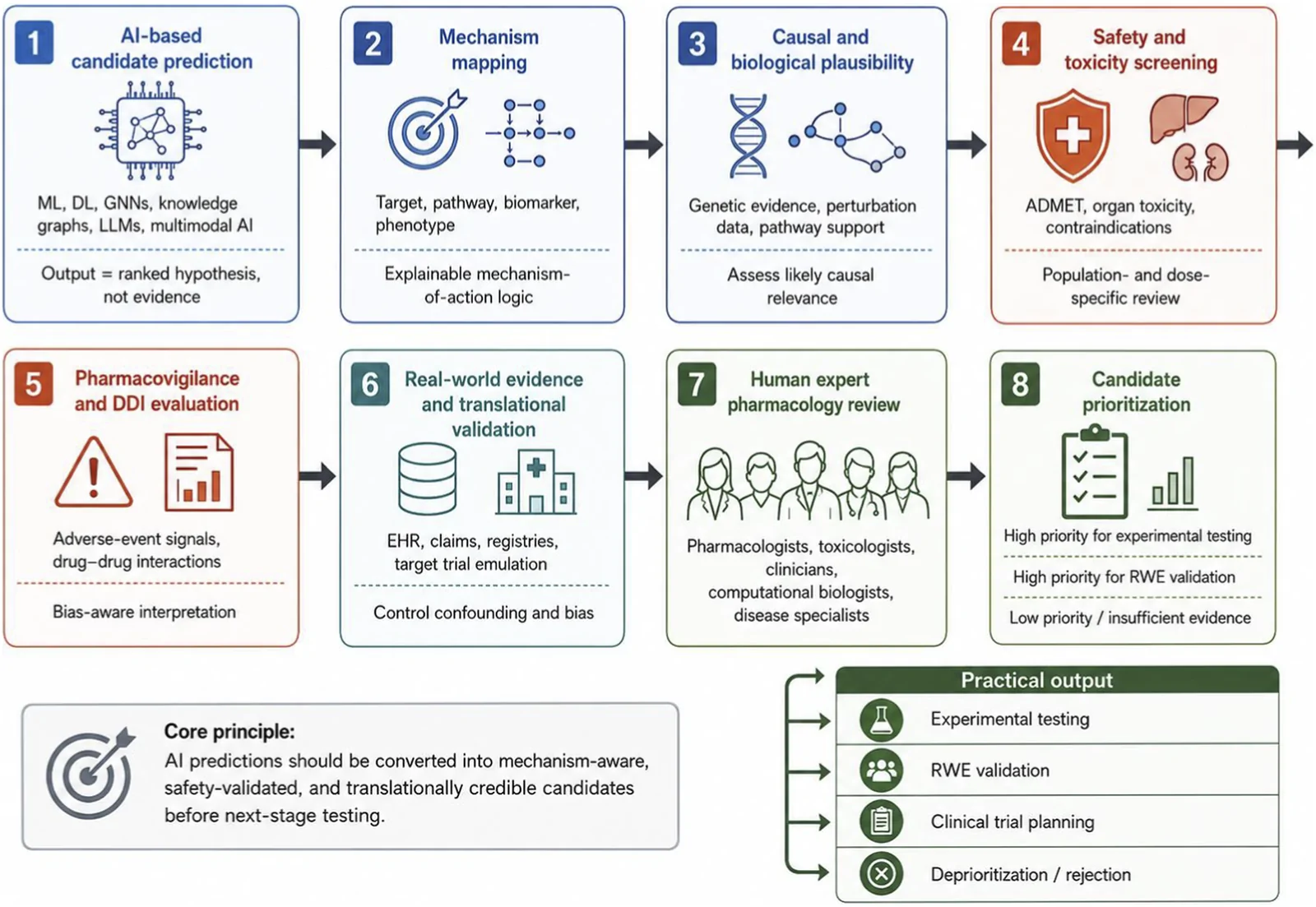
Mechanism-aware and safety-validated translational pharmacology framework.

### 7.1 Comparative positioning against existing frameworks and approaches

To better understand the scope of the contribution of the proposed framework rather than to suggest novelty on the basis of mere description, Table 6 poses selected approaches that are already available in the literature to deal with computational prediction, mechanistic explanation, real-world validation, reporting, pharmacovigilance, or regulatory credibility.

**TABLE 6:** Comparative positioning of the proposed translational pharmacology framework against existing AI-driven drug repurposing and validation approaches.

Existing framework/approach	Primary purpose	Mechanistic assessment	Safety/PV integration	Real-world/causal validation	Explicit translational decision gates	Principal distinction from proposed framework
Network medicine and population-based repurposing ((Guney et al., 2016), (Cheng et al., 2018))	Prioritize drugs using disease-network proximity and biological relationships	Partial/strong biological contextualization	Limited	Present in some studies, particularly population-level validation	No	Provides biologically informed candidate prioritization but does not integrate indication-specific safety, pharmacovigilance, causal evidence, and multidisciplinary progression criteria into a sequential workflow
Hetionet/Rephetio, KGML-xDTD and path-based KG approaches ((Jiménez et al., 2024), (Ma et al., 2023), (Himmelstein et al., 2017))	Predict drug–disease relationships and provide interpretable relational paths	Strong for relational/mechanistic traceability	Can encode adverse-event relationships but not a complete safety-validation stage	Generally limited	No	Emphasizes explainable candidate generation rather than end-to-end translational evidence convergence
TxGNN (Huaneg et al., 2024)	Zero-shot indication/contraindication prediction with interpretable multi-hop explanations	Strong mechanistic contextualization	Contraindication prediction included, but not comprehensive indication-specific safety validation	Off-label clinical alignment used as supporting evaluation	No	Integrates prediction and explanation but does not require sequential ADMET, toxicity, pharmacovigilance, causal/RWE and expert decision gates before advancement
Deep-learning/RWE trial-emulation framework (Liu et al., 2021)	Generate and test repurposing candidates using real-world patient data	Limited relative to mechanism-focused systems	Not a central integrated gate	Strong; emulated clinical trials are central	Partial	Provides patient-level effectiveness validation but does not systematically connect prediction to mechanism mapping, safety/PV assessment and multidisciplinary progression criteria
Generative-AI plus RWE validation (Yan et al., 2024)	Generate candidates with generative AI and evaluate associations in EHR datasets	Limited/incomplete	Not systematically integrated	Strong observational validation	No	Demonstrates prediction-to-RWE validation, but mechanistic, causal, safety and pharmacovigilance evidence are not integrated as prerequisite stages
TRIPOD + AI (Collins et al., 2024)	Transparent reporting of prediction-model development and evaluation	Not drug-repurposing specific	Not drug-repurposing specific	Addresses validation/reporting rather than repurposing evidence generation	No	Provides reporting standards rather than a candidate-level translational pharmacology pathway
READUS-PV (Fusaroli et al., 2024)	Transparent reporting and interpretation of disproportionality analyses	No	Strong for pharmacovigilance reporting	No therapeutic-effect validation	No	Strengthens one safety-evidence domain but does not integrate efficacy, mechanism or translational prioritization
FDA risk-based AI credibility framework (USFa and DA, 2025)	Assess AI-model credibility for a defined regulatory context of use	Context-dependent	Context-dependent	Context-dependent	Risk-based model credibility process	Defines a risk-based regulatory credibility process linking the question of interest, context of use, model risk, credibility planning, performance evaluation, documentation, and adequacy determination; it assesses AI evidence for a defined regulatory use rather than providing a complete drug-repurposing workflow
Proposed mechanism-aware and safety-validated framework	Translate an AI-generated drug hypothesis into an evidence-based next action	Explicit drug→target→pathway→biomarker/phenotype mapping plus causal plausibility	Explicit ADMET, toxicity, DDI, contraindication and pharmacovigilance stages	Explicit RWE/target-trial stage following mechanism and safety assessment	Yes—sequential evidence gates followed by multidisciplinary review and action classification	Integrates computational, mechanistic, causal, safety, pharmacovigilance, clinical and expert evidence into one candidate-level evidence-convergence pathway

The comparison helps to pinpoint the contribution of the framework. The vast majority of current computational and graph-based approaches tackle what drug to prioritize and to a lesser extent, why a model predicts to prioritize that drug. The real-world repurposing frameworks and approaches question if an association can be later identified among individual patient data, while reporting, pharmacovigilance and regulatory frameworks concern model transparency, safety-signal interpretation and context-specific model credibility. The proposed framework is different in terms of workflow architecture: it asks for evidence to be consolidated over the multiple candidate-level gates, before assigning a translational action. In particular, mechanistic plausibility is separated from causal plausibility, indication-specific safety and pharmacovigilance assessed before clinical prioritization, real-world evidence interpreted as a validation layer, and multidisciplinary expert review occurs prior to classification for experimental testing, further real-world validation, clinical development, deprioritization, or rejection. Therefore, the role of the framework is not just to include these evidence domains, but to explicitly order, interdependently connect, and tie the evidence domains to pre-established transitions.

### 7.2 Step 1: AI-based candidate prediction

The very first step is the prediction of candidates using AI. Machine learning, deep learning, graph neural networks, biomedical knowledge graphs, large language models, or multimodal AI systems could be employed at this stage to detect potential drug–disease relationships. The models can combine drug characteristics, disease genes, omics signatures, chemical structures, drug–target interactions, literature-derived relationships, electronic health records, and safety information. They can produce various things such as candidate scores, ranked drug lists, predicted indications, contraindications, or explanatory graph paths.

At this stage, candidate scores, ranked indications, predicted contraindications, and explanatory paths should be documented together with model provenance, input data sources, uncertainty information, and validation characteristics. These outputs constitute the entry point to subsequent evidence-assessment stages rather than the final translational decision. This is significant due to the possibility that AI models can be trained on partially complete, skewed, and highly curated data. Hence, candidate prediction should be considered as a starting point of the process of the translational evaluation, not its end product.

The greater the amount of relevant information provided by an AI model, the more useful its predictions become for translational interpretation. At a minimum, authors should clearly report the type of model used, the input data sources, the rationale underlying candidate ranking, the validation strategy, and the principal limitations of the model. The need for transparent reporting is due to the fact that models that are not well described are hard to reproduce or evaluate. The TRIPOD + AI reporting guidance highlights the importance of clear documentation when developing prediction models based on regression or machine learning techniques (Collins et al., 2024). While drug repurposing models are not necessarily clinical prediction models, the same principles for transparency apply to AI’s output when it is intended to inform biomedical decisions.

### 7.3 Step 2: Mechanism mapping

The second step after prediction of candidates is mechanism mapping. The next step asks: what is the likely way that the predicted drug might potentially interact with the target disease? Through a mechanism-aware assessment, the drug target, disease-relevant pathway, molecular function, biomarker connection, disease phenotype and proposed mechanism-of-action logic should be identified. This is necessary as pharmacology needs a rational relationship between the exposure of a drug, binding to the target, modulation of the pathway, and therapeutic response.

In this step, knowledge graphs and explainable AI systems are especially helpful, as they can link drugs to diseases via interpretable pathways. For instance, a candidate could be connected via a chain of events like drug, target, pathway, biomarker, phenotype, disease. This can assist pharmacologists in determining if the anticipated relationship is biologically meaningful. In particular, Hetionet/Rephetio highlighted the importance of connecting biomedical knowledge for the purpose of drug repurposing (Himmelstein et al., 2017), and KGML-xDTD and XG4Repo further underscored explanations of specific paths for making predictions in the drug–disease domain (Jiménez et al., 2024; Ma et al., 2023). Other, more recent graph foundation models like TxGNN demonstrate the potential of large-scale medical knowledge graphs for predicting indications/contraindications and citing explanatory paths (Huaneg et al., 2024).

Pharmacological explanation should be emphasized in the mechanism mapping. A technical explanation can demonstrate which features, embeddings or graph relations were used to make a prediction. A pharmacological explanation should include the mechanism of interaction of the drug with the biological target, how that target aids in the biology of the disease, whether the direction of the pathway is therapeutically appropriate, and the possibility of safety issues if the pathway is the same. So, the goal of mechanism mapping is to take the output of the model and turn it into a biologically meaningful and testable hypothesis.

### 7.4 Step 3: Causal and biological plausibility check

The third step is the causal and biological plausibility assessment. A drug–target–pathway relationship can be suggestive, but not necessarily causative. Drug-disease relationships can be discovered by AI models that are based on statistical correlations, yet these statistical relationships could be a result of common literature patterns, database-related bias, disease similarities, prescribing habit, or confounding. Thus, a candidate molecule should be considered based on the potential for a causal role in disease biology.

There are multiple types of evidence that are used to increase causal plausibility. When disease-associated variants and/or expression quantitative trait loci and/or functional genomic data suggest the same target or pathway, genetic data can provide support for the target relevance. Perturbation datasets can reveal whether the exposure to a drug alters the expression of genes, the activities of proteins, the behaviour of pathways or the phenotypes of cells in a direction that is consistent with therapeutic benefit. Pathway data can provide clues as to whether the target is associated with a disease-relevant pathway. Longitudinal clinical information could also help to formulate a causal explanation if exposure comes before the change in outcome, and the confounding has been controlled.

This is important because biological plausibility does not constitute causal proof. A target may be associated with a disease without necessarily representing a therapeutically actionable target. Similarly, disease-associated pathway alterations may not necessarily be amenable to pharmacological modulation. A biomarker could be related to outcome but not influence treatment response. Thus, future re-purposing based on AI should not be entirely based on association-based evidence; it should also consider causal evidence. When randomized trials are not available, causal interpretation can also be provided using real-world methods, such as target trial emulation (Liu et al., 2021; Hernán and Robins, 2016).

### 7.5 Step 4: Safety and toxicity screening

The fourth step comprises safety and toxicity screening. This step is designed to determine if the predicted candidate is safe enough to be considered for the new indication proposed. Safety assessment should not come after candidate ranking, as the value of an unsafe candidate, whatever computational score it has, is limited. While repurposed drugs may have well-established safety profiles, the profile could differ in another population, with a different dose, for more extended periods of time, or when combined with other treatments.

The safety screening should encompass assessment of the drug’s ADMET profile, organ-specific toxicity, the risk of drug interactions, contraindications, dose feasibility and population-specific safety. Absorption, distribution, metabolism, excretion and toxicity should be taken into account during ADMET assessment. Hepatotoxicity, cardiotoxicity, nephrotoxicity, neurotoxicity, reproductive toxicity, genotoxicity and other toxicities are considered in organ-specific toxicity screening for the intended indication. Assessing drug–drug interactions is particularly relevant when the patient population is expected to be taking multiple medications (Lee et al., 2025).

Therapeutic feasibility includes considerations of the ability to formulate and deliver the therapeutic agent to the tissue or intracellular compartment at the desired level of exposure. Degradation, insufficient cellular uptake, rapid clearance, or poor tissue distribution can hinder the ability of a biologically promising mechanism to achieve therapeutic activity, especially for macromolecular and protein therapeutics. Recently, Wang et al. have developed a biocompatible tea polyphenol nanoparticle platform to deliver protein therapeutics inside the cell. The platform ensured the protection of the protein from enzymatic degradation, preserved its activity, allowed for glutathione-triggered release from the cytosol and extended systemic circulation in experimental disease models (Wang et al., 2025), using urate oxidase as a model protein. This study does not involve the use of AI in drug repurposing but rather represents a greater translational pharmacology principle: the formulation, delivery efficiency, bioavailability, and levels of exposure achieved in the tissue or the cytosol will determine the practical feasibility of a molecularly promising therapeutic concept.

This safety step should also consider whether the proposed mechanism is likely to cause harm. A drug could alter a pathway that’s relevant to the disease, but it could also alter that pathway in normal tissues. Polypharmacology may provide therapeutic benefit through multi-target activity, but the same property can also increase the risk of off-target toxicity. Thus, safety assessment should be done in conjunction with the mapping of a mechanism—safety should not be the administrative checklist. In this context, a candidate should not be advanced to translational prioritization if there is a reasonable possibility that the candidate would be toxic, infeasible to dose, or unsafe to use in patients in the context.

### 7.6 Step 5: Pharmacovigilance and real-world safety evaluation

Pharmacovigilance and real-world safety evaluation are the fifth step. Post-marketing safety data can be valuable to understand adverse drug reactions, rare toxicities, drug–event associations, and population-level safety concerns. The spontaneous reporting systems (FAERS, VigiBase, EudraVigilance) can be used to detect adverse-event signals, which are known and unexpected and are relevant to a drug candidate being considered for repurposing.

But pharmacovigilance needs to be applied judiciously. Disproportionality analyses can provide useful safety signals, there are factors that can compromise such as missing denominator data, duplicate reports, confounding by indication, underreporting, reporting bias and missing clinical information. Thus, pharmacovigilance should not be considered as being a definitive proof of harm or safety, but as contributing to wider safety assessment. The READUS-PV guideline highlights the reporting of results from a study and the careful interpretation of disproportionality analyses, based on individual case safety reports (Fusaroli et al., 2024).

Within the proposed framework, pharmacovigilance evidence serves as a layer of a safety signal. The absence of a signal does not mean safety, and the presence of a signal does not mean that a candidate will be accepted. Rather, the findings from pharmacovigilance activities should be considered in conjunction with the drug’s ADMET predictions, toxicology data, drug labels, drug contraindications, drug–drug interaction data, patient population characteristics, and expert review. This is more relevant to translational pharmacology and is a more appropriate way of considering safety as a judgment of evidence.

### 7.7 Step 6: Real-world evidence and translational validation

The sixth step is the translation and real-world evidence. Once the mechanism and safety have been assessed, real-world data can be used to assess whether the predicted candidate has clinical support in human populations. Information on drug exposure, disease outcomes, safety events, comorbidities, and concomitant therapies may be available from electronic health records, claims databases, registries, and observational cohorts. These data are important as they represent real clinical settings, which may be larger than controlled experimental settings.

Design of real-world validation should be carefully planned. Confounding by indication, treatment selection bias, immortal time bias, exposure misclassification, outcome misclassification, missing data, and differences in baseline risk can lead to observational data being confounded. Thus, real-world data-based studies of AI repurposing should make use of suitable comparators, well-defined exposures, clinically relevant outcomes, sensitivity analysis, and application of techniques to limit the confounding. Target trial emulation is particularly useful because it involves an explicit description of the hypothetical trial protocol for the target trial, such as eligibility criteria, treatment strategies, treatment assignment, common time zero, follow-up, outcomes, causal contrasts, and analysis plan (Hernán and Robins, 2016). Eligibility, treatment assignment and follow-up should be aligned to minimize avoidable design biases; choosing an appropriate comparator and adjusting for the appropriate confounders enhance causal interpretation. In the proposed approach, the use of target trial emulation should thus be seen as a structured design for causal validation in the real world, and not as another predictive modelling method.

Liu et al. (2021) illustrated the potential of using deep learning models and real-world patient information to simulate clinical trials for testing drug repurposing candidates. The value of such an approach is that it takes the evaluation of candidates beyond computational ranking to patient-level evidence (Liu et al., 2021). Nevertheless, in the real world, the evidence must be viewed as supportive, rather than confirmatory, until the design is robust, confounding is minimized and results are consistent between datasets and/or methods.

### 7.8 Step 7: Human expert pharmacology review

Human expert pharmacology review is the seventh step. Although AI systems can synthesize data and generate hypotheses, expert interpretation is required before candidates are prioritized for experimental, real-world, or clinical evaluation. The multidisciplinary review should feature clinical pharmacologists, toxicologists, computational biologists, disease specialists and, if applicable, regulatory specialists.

Four key areas should be assessed during expert review–mechanism, safety, feasibility and clinical relevance. Mechanism review includes the question of the biological plausibility and testability of the drug–target–pathway–disease logic. Safety review questions: Is there an acceptable level of toxicity, contraindication, dose feasibility, and drug–drug interactions in the target population? Feasibility review involves determining whether the candidate could be delivered and applied in the intended setting realistically, such as in the formulation/delivery system, route of administration, expected bioavailability and target tissue exposure, dosage, availability, monitoring requirements, and duration of treatment. The clinical relevance review question is whether the candidate addresses an unmet need and whether the benefit that is expected would justify continued investigation.

For consistency and transparency, the multidisciplinary review can be done on a pre-established candidate level assessment form that addresses each of the four domains described above: mechanism, safety, feasibility, and clinical relevance. It is important to assess the candidate individually by each member of the panel, to reduce the influence of the group. There are three levels of ordinal scores for each domain: 0 = insufficient evidence or major concern; 1 = partially supportive evidence or substantial uncertainty; and 2 = sufficiently supportive evidence for progression to next stage. The reviewer should include a concise, evidence-based explanation for each grade and highlight key uncertainties and conflicting results as well as missing information. The domain scores obtained are not to be used as validated regulatory thresholds and they are not a simple numerical ranking system; rather, the scores are meant to be used to organize multidisciplinary judgment.

After an independent assessment the panel should discuss domains in which there are significant differences in scores or a reviewer finds there is a clinically important concern. Special consideration should be given to issues of toxicity, contraindications, drug–drug interactions, dose and/or exposure feasibility, and the direction of the proposed biological mechanism, as these should not be discounted solely based on higher scores in other evaluations. Evidence-based consensus is the preferred method of decision making. In the event of a disagreement, the reason for the disagreement, supporting evidence and minority opinion should be recorded explicitly and the candidate should normally be classified as needing supplemental evidence rather than advanced on the basis of an averaged score alone. When a disagreement is beyond the expertise of the original panel, additional pharmacology, toxicology, disease-specific, statistical, or regulatory expertise may be needed.

The structured review should then generate domain scores, as well as a documented rationale, and a recommended disposition (e.g., experimental testing, further real-world testing, clinical-development consideration, deprioritization, insufficient evidence). This allows for human oversight of concerns not captured in the AI-generated content such as ethical, patient acceptability, unusual contraindications, monitoring needs, and regulatory constraints.

### 7.9 Step 8: Candidate prioritization for next-stage testing

The last step is to prioritize the candidates. Evidence gathered over the course of the framework helps stakeholders decide on next steps. The results should not be a ranked list of drugs. Instead, the candidates should be sorted by the strength of the mechanism, safety of the mechanism, causal plausibility, real world support, and feasibility.

The structured domain assessments from Step 7 are used to achieve operationalisation of candidate prioritization in Step 8. Mechanism, safety, feasibility and clinical relevance can be scored 0–2 for each candidate, giving a maximum total score of 8 points. These are suggested thresholds that can be used as practical decision criteria for the framework and not as validated regulatory thresholds. More importantly, there is no “compensation” for major safety issues in the aggregate score, that is, the exposure should not be advanced if the total score is good but the toxicity, contraindication, drug–drug interaction or clearly unfeasible therapeutic exposure is clinically unacceptable.

The candidates can then be placed in four operational categories. High priority candidates tend to score 6–8 overall, with no outstanding major safety concern and no domain that scores 0 as a result of substantive adverse evidence. Of those candidates, ones with a biologically plausible and testable mechanism but without direct mechanism-aligned experimental confirmation are candidates who are given high priority for experimental testing. In contrast, candidates are seen as high priority for real-world validation when there is a sufficient amount of human exposure and relevant clinical data to meaningfully assess candidates’ products through observation, yet there is insufficient causal or indication-specific clinical data. This difference in overall numerical rank is not a difference in the distinction between these two high priority categories, but rather only represents the key remaining evidence gap.

Candidates with an overall score of 5 or lower should be generally considered low priority due to weak and/or adverse evidence or a major concern in a mechanistic, safety or feasibility area despite having strong evidence in other domains. An insufficient-evidence classification should be reserved for when poor scores are primarily due to the lack of evidence, evidence that is conflicting, or evidence that is uninterpretable, rather than evidence that the candidate is not capable of performing the task. For candidates who have two or more domains that are insufficiently supported due to missing or contradictory evidence or when there is unresolved disagreement between multiple disciplines after Step 7, it is recommended to continue to classify them as insufficient evidence until they receive more information. Low priority refers to evidence that contradicts advancement and insufficient evidence means evidence that does not allow for a reliable advancement decision to be made.

These thresholds offer a clear linkage between structured multidisciplinary evaluation and the next step in translation, and retain expert judgment. The aggregate score should therefore only be read in conjunction with evidence in the domains and safety constraints and with regard to the reasons for the low or missing score, but not as an automatic ranking criterion.

If there is contradictory evidence from different stages of the framework, the conflicting results should be not averaged or selectively interpreted in favor of the initial AI prediction. Smaller emphasis should be placed on indirect and less specific evidence, on evidence that was not independently validated, and on evidence that is not methodologically sound. Advancement should be avoided in the face of major safety concerns or convincing evidence of harm, even if there is computational or mechanistic evidence that suggests it may be safe; robust negative experimental or clinical evidence should not be overcome by positive computational predictions. If there is evidence of similar quality that is still contradictory, the candidate should be considered insufficient evidence and asked to complete further validation specifically aimed at settling the disagreement. The source, direction and intensity of the competing evidence should be recorded prior to final prioritization.

The proposed framework culminates in this staged prioritization, which is the endpoint of the process. This staged approach supports progression from computational prioritization to evidence-based translational decision-making by emphasizing pharmacological rationale, safety, causal plausibility, and feasibility rather than computational ranking alone. Table 7 presents an overview of the operational evidence and decision logic and its respective elements in the framework.

**TABLE 7:** Operational components of the proposed translational pharmacology implementation framework.

Framework step	Decision question	Evidence domains to be considered	Literature basis and author-proposed operational role
Prediction	Why was this drug prioritized?	Model output, candidate score/ranked indication, input-data provenance, validation summary and uncertainty information where available	**Literature basis:** AI/ML candidate ranking and transparent model reporting are established ((Vamathevan et al., 2019), (Collins et al., 2024)). **Author-proposed role**: prediction is treated as hypothesis generation and cannot independently justify translational advancement
Mechanism mapping	How could the drug alter disease biology?	Drug–target–pathway–disease relationship, mechanism-of-action logic, biomarker/pathway support and expected direction of modulation	**Literature basis:** network medicine, knowledge graphs and mechanism-oriented approaches support biological contextualization and explainability ((Perdomo-Quinteiro and Belmonte-Hernández, 2024; Huaneg et al., 2024; Jiménez et al., 2024; Ma et al., 2023), (Guney et al., 2016; Cheng et al., 2018; Himmelstein et al., 2017)). **Author-proposed role:** these relationships are organized into an structured pharmacological evaluation step
Causal plausibility	Is the proposed relationship plausibly causal rather than only associative?	Genetic evidence, Mendelian randomization, perturbation data, functional/pathway evidence, longitudinal evidence and appropriately designed causal analyses	**Literature basis:** genetic evidence, Mendelian randomization, perturbational and functional studies, and target-trial approaches can strengthen causal interpretation ((Liu et al., 2021), (Schmidt et al., 2020), (Hernán and Robins, 2016)). **Author-proposed role:** causal plausibility is assessed separately from mechanistic association
Safety screening	Is the candidate sufficiently safe and dose-feasible in the proposed new context?	ADMET, organ-specific toxicity, formulation and delivery feasibility, bioavailability and dose/exposure feasibility, contraindications, DDI, and population-specific risk	**Literature basis:** ADMET, toxicology and DDI assessment are established pharmacological considerations ((USFa and DA, 2025), (Lee et al., 2025), (Zhang et al., 2025)). **Author-proposed role:** safety is evaluated before translational prioritization rather than only after efficacy-focused ranking
Pharmacovigilance	Are relevant post-marketing adverse-event signals present?	Bias-aware analysis of spontaneous-reporting systems together with labels and complementary safety evidence	**Literature basis:** spontaneous-reporting systems and disproportionality analysis are established pharmacovigilance tools ((Fusaroli et al., 2024), (Vogel et al., 2020)). **Author-proposed role:** pharmacovigilance is used as a supporting safety domain; presence or absence of a signal is not treated as proof of harm or safety
Real-world validation	Is there clinically relevant support in patient populations?	EHRs, claims data, registries, observational cohorts, active-comparator analyses, sensitivity analyses or target-trial emulation	**Literature basis:** RWE and trial-emulation approaches have been applied to drug repurposing and observational treatment-effect assessment ((Liu et al., 2021), (Yan et al., 2024), (Hernán and Robins, 2016)). **Author-proposed role:** RWE follows mechanism and safety assessment and contributes supportive rather than automatically confirmatory evidence
Expert review	Is the candidate pharmacologically, clinically and operationally credible?	Structured multidisciplinary assessment of mechanism, safety, feasibility and clinical relevance	**Literature basis:** transparent reporting, human oversight and context-of-use evaluation are established in biomedical/regulatory AI assessment (Collins et al., 2024); (DiFrancesco, 2026). **Author-proposed role:** multidisciplinary assessment is positioned as an explicit gate before final prioritization
Prioritization	What should be the next translational action?	Integrated consideration of Step 7 domain scores together with computational, mechanistic, causal, safety, real-world and feasibility evidence, including missing, conflicting or discordant findings	**Literature basis:** Candidate ranking and prioritization are established in computational repurposing. **Author-proposed role:** Step 7 scores guide final prioritization. Candidates scoring 6–8, with no score of 0 or major safety concern, are high priority; those scoring ≤5 due to weak or adverse evidence are low priority, while substantial missing or conflicting evidence indicates insufficient evidence. Major safety or feasibility concerns override the total score. These thresholds are proposed decision aids, not validated regulatory cut-offs

The framework represents an author-proposed evidence-integration model and does not define validated regulatory thresholds or approval requirements.

Bold values indicate the highest score/ranking or the most favorable outcome among the evaluated candidates/stages within the corresponding comparison category. These values are highlighted to facilitate identification of the strongest evidence or priority level.

This staged prioritization is a means of putting the above comparativeness distinction into practice. Instead of the workflow ending with a candidate’s score or an explanatory path, evidence gathered is converted into the next action in the workflow. Thus, while a candidate with computational promise could still be at a very early stage in development if the mechanistic, causal, or safety evidence is not sufficient, a candidate with evidence from multiple independent evidence domains may progress to experimental testing, full-scale real-world testing, or clinical-development planning. The framework then uses a decision on ‘translational readiness’ as an evidence-convergence decision, not a count of model rank.

Table 7 summarizes the specific functions of each stage and the progression questions for each stage. The ability of the candidate to be evaluated is supported by computational support, the hypothesis is biologically credible based on evidence derived from the mechanism, the hypothesis is not incompatible with safety considerations and pharmacovigilance data, the hypothesis is not impossible given real-world evidence, and expert review combines all of these data to support a translational decision. This is different from frameworks where the assessment of these evidence domains is performed separately or without a progression rule.

### 7.10 Detailed case-based appraisal of the proposed translational framework

Two contrasting cases were selected to examine how the proposed framework operates across different levels of translational maturity. Baricitinib for COVID-19 is a relatively mature case in which the repurposing hypothesis, supported by artificial intelligence, advanced from mechanistic studies, experimental testing, randomized clinical trials, safety testing, and regulatory approval. In contrast, generative-AI prioritization in Alzheimer’s disease has progressed to a much earlier translational phase where computational prioritization of candidates was followed by multi-database real-world validation, but mechanistic, causal, safety and prospective clinical evidence were incomplete. The cases are not presented as examples of successful re-purposing, but rather to determine if the framework is capable of separating a candidate from the other cases that has convergent evidence from those that are still clinically interesting, but are under less validation.

#### 7.10.1 Baricitinib for COVID-19: from AI-guided hypothesis to clinical translation

Baricitinib provides one of the clearest examples of AI-assisted drug repurposing progressing from computational prioritization to mechanistic and clinical validation. Originally developed and approved as a Janus kinase (JAK)1/JAK2 inhibitor for rheumatoid arthritis, baricitinib was identified early in the COVID-19 pandemic using an AI-enhanced biomedical knowledge-graph approach as a potential therapeutic candidate. The computational rationale was unusual because it proposed a dual mechanism: established JAK1/JAK2 inhibition could suppress excessive inflammatory cytokine signaling, whereas predicted inhibition of numb-associated kinases, including AAK1 and GAK, could potentially interfere with host-mediated viral entry and propagation (Richardson et al., 2020). In this instance, the role of AI was to speed up the process of hypothesis generation, by linking existing pharmacology to disease-relevant host pathways that could be tested experimentally. This distinction is central to the proposed framework: computational novelty can justify prioritization, but progression depends on whether the predicted biological relationship survives independent pharmacological and clinical testing.

Importantly, this computational hypothesis was subsequently subjected to pharmacological validation rather than being treated as sufficient evidence. Experimental studies confirmed baricitinib activity against AAK1, BIKE and GAK at nanomolar concentrations, demonstrated inhibition of cytokine pathways implicated in COVID-19, and showed reduced SARS-CoV-2 infectivity in primary human liver spheroids at clinically relevant exposures (Stebbing et al., 2020). Clinical validation then progressed through randomized controlled trials. In ACTT-2, 1,033 hospitalized patients were randomized to baricitinib plus remdesivir or remdesivir plus placebo; median recovery was 7 versus 8 days, respectively, with particularly pronounced improvement among patients receiving high-flow oxygen or non-invasive ventilation (Kalil et al., 2021). The next COV-BARRIER phase three trial needs to be interpreted more carefully. The absolute risk difference between the baricitinib group and the placebo group was −2.7 percentage points (95% CI −7.3 to 1.9) for the prespecified composite primary endpoint of progression to high-flow oxygen, non-invasive ventilation, invasive mechanical ventilation/ECMO or death by day 28 (Marconi et al., 2021). As a consequence, COV-BARRIER failed to achieve its primary efficacy endpoint and it was not possible to rule out a potential lack of efficacy for the predefined disease-progression endpoint. This is a negative primary result but is methodologically important and should not be placed on a pedestal as a positive result to be presented later. The prespecified multiplicity-testing procedure resulted in non-statistical significance of the primary analysis; therefore, the key secondary outcomes should not be considered formally statistically significant after multiplicity adjustment. The difference in mortality between baricitinib and placebo observed over 28 days (8% versus 13%, respectively; HR 0.57, 95% CI 0.41–0.78) is therefore also a secondary, clinically important but not statistically significant result, and not evidence of the drug’s potential to turn around the negative primary endpoint. Therefore, the correct interpretation is one of discrepant trial evidence - no significant reduction in the prespecified progression endpoint, but a strong mortality signal that must be taken into account with the rest of the trial evidence and not in isolation (Marconi et al., 2021). Baricitinib was subsequently approved by the US FDA in May 2022 for certain hospitalized adults with COVID-19 requiring supplemental oxygen, non-invasive or invasive mechanical ventilation, or extracorporeal membrane oxygenation (Food, 2022).

In the proposed framework, baricitinib is a representation of an ongoing process of evidence convergence, not a single successful prediction based on AI. Step 1 (candidate prediction) – AI-guided prediction of baricitinib’s activity against kinases associated with numb that mediate viral entry, based on the known JAK1/JAK2 pharmacology. Step 2 (mechanism mapping) was enhanced by the connection of the hypothesized dual pharmacology with both inflammatory JAK signaling and AAK1/BIKE/GAK-host cell-associated processes. Experimental evidence of causal and functional plausibility was further provided in Step 3, which included evidence of kinase inhibition and modulation of inflammatory signaling, and reduced viral infectivity at clinically relevant exposures (Stebbing et al., 2020). This progression is crucial as the computer-generated mechanism was translated to an experimentally measurable molecular and phenotypic effect instead of just being an explanatory graph relationship.

The latter stages also demonstrate how one cannot assume that a mechanism is sufficient to indicate translational readiness. In Step 4 (safety and dose feasibility), some pre-existing pharmacokinetic and safety data from RA were found to be applicable, but this data did not apply directly to acutely ill patients with COVID-19 receiving different background treatments. Thus, efficacy and safety evidence for indication-specific effects were also provided by the randomized ACTT-2 and COV-BARRIER trials (Kalil et al., 2021; Marconi et al., 2021). COV-BARRIER also shows the need to incorporate negative and statistically conflicting results into evidence convergence as well as the need to treat clinical validation as a binary success/failure step. The trial did not show a statistically significant advantage for this prespecified primary disease-progression endpoint, and this result impacted the inferential status of the subsequent key secondary endpoints as per the multiplicity-testing procedure used in the trial. Therefore, the benefit in the number of deaths is a helpful supporting message, rather than a definitive positive trial. In the framework proposed, this pattern would take into account the mechanistic support, the negative primary clinical endpoint, the clinically important nominal mortality signal, the acceptable trial safety profile, and all of these would be explicitly reconciled. Such a profile could justify further evaluation and integration of evidence, but shows why decisions regarding translation should not be made based on selective focus on whichever endpoint is most favorable.

The post-marketing safety information, clinical evidence, multidisciplinary interpretation, and a defined progression decision would be needed for steps 5–8. Subsequent regulatory authorization and approval show that the candidate eventually went well beyond computational prioritization (Food, 2022), but this case study should not be considered as prospective validation of the proposed framework. On the contrary, the evidence trajectory of an AI-supported repurposing candidate that made it to clinical and regulatory use, despite its mixed clinical-evidence profile, is quite similar to what the framework recommends for convergence across mechanism, exposure, safety, and clinical outcomes, as shown in Table 8.

**TABLE 8:** Review-derived application of the proposed translational framework to the baricitinib and Alzheimer’s disease case studies.

Framework stage	Baricitinib for COVID-19	Generative-AI candidates for Alzheimer’s disease	Case-based interpretation
1. Candidate prediction	AI-assisted knowledge integration prioritized baricitinib based on JAK inhibition and predicted AAK1/GAK-related host-pathway effects (Richardson et al., 2020)	Repeated GPT-4 querying generated 59 unique candidates across ten runs; the ten most frequent outputs were selected for EHR evaluation. Frequency of generation was used as a heuristic and was not a validated measure of therapeutic probability (Yan et al., 2024)	Candidate generation was present in both cases, but its evidentiary basis differed. For the AD study, stochastic repeated querying and frequency-based selection constitute an exploratory, non-validated hypothesis-generation method; downstream EHR findings should not be interpreted as retrospective validation of that selection heuristic
2. Mechanism mapping	Dual anti-inflammatory and host-cell-entry rationale was defined and subsequently investigated experimentally (Richardson et al., 2020); (Stebbing et al., 2020)	Detailed candidate-specific drug–target–pathway mechanisms were not integrated into the clinical validation workflow (Yan et al., 2024)	Mechanistically developed for baricitinib; incompletely addressed for the AD candidates
3. Causal/functional plausibility	Kinase activity, inflammatory signalling, viral infectivity and clinically relevant exposure were experimentally evaluated (Stebbing et al., 2020)	Observational associations were demonstrated, but causal molecular mechanisms were not established ((Yan et al., 2024)	Functional evidence strengthens baricitinib; AD findings remain primarily associative
4. Safety and dose feasibility	Pre-existing RA pharmacology informed evaluation, followed by COVID-19-specific safety assessment in randomized trials (Kalil et al., 2021), (Marconi et al., 2021)	Systematic indication-specific dose, ADMET, toxicity and DDI assessment was not integrated into candidate selection (Yan et al., 2024)	More extensively addressed for baricitinib
5. Pharmacovigilance	Existing/post-marketing safety knowledge could contribute to candidate assessment, although pharmacovigilance was not the basis of the original AI prediction	No dedicated pharmacovigilance stage reported in the generative-AI validation workflow	Neither case should treat spontaneous safety information as proof; systematic integration is more clearly missing from the AD workflow
6. Real-world/clinical validation	Experimental evidence followed by randomized clinical trials; Randomized clinical evidence was mixed: ACTT-2 supported improved recovery with baricitinib plus remdesivir, whereas COV-BARRIER did not meet its prespecified primary disease-progression endpoint; COV-BARRIER nevertheless showed a clinically important mortality signal that was nominal because the primary endpoint failed under the multiplicity-testing hierarchy (Kalil et al., 2021); (Marconi et al., 2021)	Propensity-score-matched EHR analyses across VUMC and All of Us provided replicated observational associations (Yan et al., 2024)	Evidence type differs materially: randomized clinical evaluation versus observational RWE
7. Multidisciplinary/regulatory review	Progressed to regulatory decision-making and clinical use (Food, 2022)	No progression to equivalent regulatory or prospective clinical decision reported	Translational maturity differs markedly
8. Next-stage prioritization	Evidence convergence supported progression from hypothesis to clinical evaluation and regulatory use	Candidates remain appropriate for mechanistic, causal, safety and prospective validation rather than therapeutic claims	Framework distinguishes a mature translated case from an intermediate hypothesis-validation case

This table represents the authors’ retrospective application of the proposed framework to the cited cases and is not a validated scoring system or formal assessment performed in the original studies. “Addressed” or “incompletely addressed” refers only to whether the corresponding evidence domain was reported in the cited case literature.

#### 7.10.2 Generative-AI prioritization for Alzheimer’s disease: real-world validation without causal confirmation

A complementary example is provided by Yan et al. (2024), who evaluated whether a generative AI system could prioritize existing drugs for repurposing in Alzheimer’s disease (AD). GPT-4 was queried repeatedly to generate candidate drugs, producing 59 unique candidates across ten independent runs; the ten most frequently suggested drugs were subsequently evaluated in electronic health-record data from Vanderbilt University Medical Center and the NIH All of Us Research Program (Yan et al., 2024). The investigators did not accept the generative output as evidence of therapeutic benefit but subjected it to observational clinical validation using propensity-score-matched cohorts and Cox proportional-hazards models.

The candidate-generation stage itself, however, warrants substantial methodological caution. Repeated querying of a general-purpose generative model is not equivalent to a validated drug-ranking algorithm, because the frequency with which a drug is generated is neither a calibrated probability of therapeutic efficacy nor an established pharmacological prioritization metric. Yan et al. explicitly acknowledged that the number of times a candidate appeared across ChatGPT queries might not correspond to its therapeutic promise (Yan et al., 2024). Moreover, the most frequently generated candidates were largely drugs already discussed extensively in the Alzheimer’s disease literature, raising the possibility that generation frequency reflected literature salience or representation in the model’s training corpus rather than discovery of previously unrecognized pharmacological relationships. The production of 59 unique candidates across only ten independent runs further demonstrates output instability, while candidate selection remained sensitive to prompt formulation, model version, stochastic generation, and the opaque composition of the model’s training data. Consequently, repeated GPT-4 querying should be regarded as an exploratory hypothesis-generation heuristic rather than a reproducible or validated candidate-selection method. Its output requires independent evidence before it can meaningfully satisfy the candidate-prioritization stage of a translational workflow.

In the VUMC dataset, metformin, losartan and minocycline were associated with significantly lower incident AD risk, while meta-analysis across the two clinical datasets supported protective associations for metformin, simvastatin and losartan. Metformin produced the strongest and most consistent signal, with a meta-analytic HR of approximately 0.67, corresponding to a 33% lower observed risk of incident AD among exposed individuals (Yan et al., 2024). However, this example also demonstrates why real-world association should not be equated with successful therapeutic repurposing. The authors themselves identified residual and unmeasured confounding, incomplete EHR information, exposure misclassification, limited statistical power for less frequently used drugs, and the inability of the observational design to establish causal effects or mechanisms.

The Alzheimer’s study has a very different translational status compared with the example of baricitinib, as seen from the proposed framework. It is therefore only provisionally fulfilled to Step 1 (candidate generation). A set of candidates was iteratively culled using GPT-4 queries and then prioritized based on frequency, but there was neither a validated pharmacological scoring function, nor a candidate universe, nor an experimentally calibrated model to produce the frequency-based prioritization. Ten runs generated 59 distinct outputs, showing that candidate generation was stochastic, and the top ten of these drugs were those appeared most often in the list, without any evidence that they were the most likely to be of therapeutic value. Indeed, the investigators at the start realized that query frequency may not necessarily directly reflect therapeutic potential (Yan et al., 2024). In the suggested system, such generative production would then warrant further screening but would not have a strong evidentiary weight until confirmed by independently derived mechanistic, pharmacological or clinical evidence. The ten most common candidates were also examined in two different clinical data environments in the context of propensity score matched cohorts and time-to-event analysis in Step 6 (real-world validation) (Yan et al., 2024). The cross-dataset evaluation is more robust evidence than the original GPT-4 generation frequency, but still does not validate the generative candidate-selection method itself in a retrospective fashion.

But in the real world, signal replication does not equal therapeutic validation. In the case of metformin, for instance, the meta-analytic relationship between the drug and a reduced incidence of AD is an indicator that warrants further investigation, but does not specify which drug–target–pathway relationship was responsible for the observed association, whether causal or not, nor whether treatment specifically for AD would show the same effect. Although the design is based on observational studies, the original study recognized that the study was still prone to residual confounding, exposure misclassification, incomplete clinical data, treatment selection, and other differences between the exposed and the comparator groups (Yan et al., 2024). In consequence, the mechanistic mapping and causal plausibility (Steps 2 and 3) were not adequately covered, nor was the integration of pharmacovigilance aspects in the candidate validation process (Steps 4 and 5) integrated into the candidate-validation workflow.

In the proposed framework, these candidates would not be enough, however, to be considered high-confidence candidates for clinical prioritization simply because they were repeatedly generated by GPT-4 and linked to lower AD incidence in observational datasets. Follow up rational testing, using mechanistic and perturbational methods for individual candidates followed, if applicable, by more robust causal analysis, target-trial emulation, indication-specific safety assessment and eventually prospective clinical assessment would be a logical next step. This is because the Alzheimer’s case is instructive not for the fact that the generative AI was able to discover an existing therapy for Alzheimer’s but because this example shows how to narrow its AI-generated hypotheses with independent clinical data while maintaining a clear margin above the evidence threshold required for therapeutic claims, as shown in Table 8.

The difference between the two cases is instructive, starting with a set of hypotheses generated computationally in both, and differing in the extent and nature of the downstream evidence. Baricitinib collected mechanistic, causal, and indication-specific safety evidence as a result of these studies, while the Alzheimer’s candidates moved from the generation of hypotheses to observational validation, without any mechanistic or causal safety evidence. The framework therefore does not make a distinction between the sophistication of the AI model producing the candidate, but rather between the completeness and convergence of evidence that comes after the candidate.

## 8 Discussion

Combined, the literature examined indicates that the question of what evidence should be used to advance a candidate is not one of candidate generation, but of the level and type of evidence for advancement. The proposed framework proposes to overcome this issue by making progression decisions clear and measurable and ensuring that uncertainty, negative findings and discordant evidence are not erased, but rather retained, both as part of the decision-making process and in an audit trail. It is, therefore, mainly organizational and decisional, connecting evidence being generated in different computational, pharmacological, safety and clinical workflows to defined, translational actions.

### 8.1 Key controversies and schools of thought in AI-driven repurposing

The present contentious arena of AI-driven drug repurposing is defined by several key debates. The first controversy concerns the criterion by which an AI-prioritized candidate should be judged. Computational approaches emphasize benchmark performance, AUC, precision, recall, link prediction, and ranking quality, whereas pharmacology-centred assessment emphasizes target engagement, pathway directionality, exposure feasibility, safety, and clinically meaningful effects (Zhou, 2021). These perspectives are complementary rather than interchangeable: computational metrics characterize model discrimination, whereas pharmacological criteria address therapeutic plausibility.

The second debate concerns the trade-off between predictive performance and explainability. Although deep learning, graph neural networks, and foundation models can identify complex and non-obvious drug–disease relationships, their internal reasoning may be difficult to interpret, creating tension between predictive accuracy and pharmacological transparency. Explainability of the model can be achieved with feature importance, embedding, attention weights scores or graph scores, but can often be irrelevant for the pharmacologist or clinician. The explanation should be pharmacologically actionable and include an explanation about the interaction between the drug and the target, the relationship between the target and the biology of the disease, the direction of pathway modulation (i.e., if the pathway is stimulated, should the drug stimulate it, and *vice versa*), and if the pathway is stimulated, are there safety concerns with the same mechanism of action? Addressing this can be challenging with knowledge graphs and path-based explanations, though even an interpretable path does not imply causality or clinical efficacy (Jain and Wallace, 2019; Schenone et al., 2013).

A third issue in drug repurposing is regarding the safety assumptions. Repurposing drugs is generally portrayed as being quicker and less risky than *de novo* drug discovery because drug candidates may have clinical exposure, pharmacokinetic data, and safety data (Pu et al., 2019). But the safety of a new indication, disease context, dose, duration, route or patient population cannot be guaranteed just because it has been approved earlier. A drug that is safe in one therapeutic context could be unsafe in a different group of patients having different comorbidities, organ function, immune status, or co-medication pattern. Safety should thus not be a last phase, following computation prioritization. Rather, integration of ADMET assessment, organ-specific toxicity, contraindication review, drug–drug interaction screening, pharmacovigilance evidence and population-specific safety evaluation should be done early on.

The fourth debate is about the place of real-world evidence. To assess whether the pattern of clinically relevant outcomes in patient populations of AI prioritized candidates is detectable in electronic health records, claims databases, registries, and observational cohorts can be used. In the real world, however, there are several potential sources of confounding, including immortal time bias, baseline risk differences, missing data, exposure misclassification, outcome misclassification, and treatment selection bias. Real-world evidence should therefore neither be dismissed nor overinterpreted. Its value is greatest when generated using rigorous designs, appropriate comparators, sensitivity analyses, and causal-inference frameworks such as target-trial emulation (Liu et al., 2021; Hernán and Robins, 2016). So, clinical evidence alone is not sufficient to validate efficacy and should be considered a single supporting step in the process of translational support.

The fifth debate relates to the reliability thresholds that foundation models and large language models need to surpass to meaningfully contribute to the field of translational pharmacology. They are capable of the literature synthesis, biomedical reasoning, and generation of hypotheses that goes beyond hallucination, but they also have significant limitations. Present systems can make unsupported mechanistic claims, have imperfectly calibrated uncertainty, and have variable output in answer to repeated or linguistically altered inputs (Farquhar et al., 2024; Language Models et al., 2024). Yet, careful interpretation of benchmark performance is also necessary since the accuracy of the data may differ from expectations due to data contamination and limited robustness under novel biomedical conditions (Language Models et al., 2024; North American et al., 2024). The limitations are especially relevant in the context of drug repurposing, for example, if the inference to the target, pathway, safety, or dose is wrong, then the downstream prioritization of candidates may be impacted. In addition to generic benchmark performance, regulatory usage adds a second requirement for credibility: model performance should be assessed based on the context of use, risk, documentation, validation and lifecycle management of the model (Agency, 2024; DiFrancesco, 2026). Therefore, foundation models should complement, not supplant, the work of pharmacologists, and output from these models should be verified for evidence, assessed for uncertainty, mechanistically validated, evaluated for safety and subject to human oversight prior to prioritization for translation.

### 8.2 Practical recommendations for future AI-driven repurposing studies

These principles should be tested in the future through a prospective study rather than another set of candidate rankings. Progression criteria and candidate sets should be prespecified; findings should be reported in a transparent manner; the model and data provenance should include evidence of the model’s development; and decisions for progression should be made based on independent experimental, clinical, or real-world findings. Comparative studies are especially required to assess whether mechanism-aware and safety-constrained workflows are more efficient, reproducible, and correctly calibrated in the selection of candidates, compared to prediction-focused workflows.

### 8.3 Future directions

The development of AI-driven drug repurposing will require multi-modal, causal, explainable and safety-constrained systems in the future. Future multimodal systems should integrate chemical structure, drug–target interactions, omics profiles, pathway information, biomedical literature, clinical-trial data, electronic health records, and pharmacovigilance data within a unified translational framework. Such integration may improve candidate discovery by combining complementary molecular, biological, clinical, and safety information.

Beyond the performance of the AI model and the need for biological validation, system-level conditions play a vital role in the translation of AI-enabled biomedical innovation. For instance, a cross-domain example was provided by Hu et al. on the development of rehabilitation assistive technology transfer networks in the Yangtze River Delta and its findings that technology-transfer activities spread into a more multicenter network and that coordination between public-health systems and technology transfer also increased over time. The connection between AI inventive capacity and technology transfer was, however, still relatively less developed, and this indicated that there was still a lag between technological innovation and actual technology diffusion (Hu et al., 2026). While this work is not about drug repurposes, it demonstrates a general rule for the successful introduction of AI into biomedical innovations: institutional capacity, funding, standards, infrastructure, inter-organizational transfer networks, and public-health needs are all crucial for success. These considerations should be kept in mind when implementing AI-powered therapeutic innovations from research settings to clinical and population-level implementation.

Causal AI and target trial emulation are also future directions that are significant as the field needs to progress beyond association-based prediction to evidence that can be used in clinical decision-making and trial planning. Causal approaches can aid in identifying whether the drug–disease association is due to a plausible therapeutic mechanism, or if it is just a statistical association. While foundation models and large language models can facilitate hypothesis formulation and knowledge consolidation, they can be limited by a curated pharmacological knowledge, safety constraints, explicit referencing, and expert oversight. Future prospective studies should compare alternative AI prioritization strategies using predefined translational endpoints and determine whether mechanism-aware and safety-constrained models improve candidate-selection efficiency over prediction-focused approaches. The generation of regulatory-ready evidence should be a major objective, and the AI output should be transparent, auditable, reproducible, interpretable, and must be tied to the mechanism(s) of action, safety evidence, and clinical context.

### 8.4 Limitations of this review

There are a number of limitations in this review. The evidence was organized into four key areas (computational, mechanistic, safety, real-world, and regulatory), and a structured critical review was adopted; however, significant heterogeneity in disease area, data source, AI architecture, validation strategy, and endpoints made the pooling of evidence and direct meta-analysis of model performance impractical. Additionally, the dynamic nature of graph foundation models, multimodal AI, and large language models leads to some new methods lacking independent or prospective validation. Moreover, the evidence and gaps found during the review of the literature has resulted in a synthesis of the evidence and a framework that has not yet been prospectively validated as a formal candidate selection instrument. Future studies should thus examine its reproducibility, inter-rater reliability, predictive value and applicability to other disease areas and repurposing settings.

## 9 Conclusion

The ability to generate drug-repurposing hypotheses has been greatly extended by AI, with inconsistent downstream validation. This review suggests a structured evidence-integration framework to map computational hypothesis to specific next stage decisions while maintaining uncertainty and conflicting or invalid evidence. The framework needs to be assessed in prospective studies to see if it enhances the reproducibility and efficiency of candidate selection in other repurposing contexts.
