# Correction: Mechanism-aware and safety-validated AI for drug repurposing: a critical review and translational pharmacology framework

**DOI:** 10.3389/fphar.2026.2009326

**Published:** 2026-10-12

**Authors:** Moumita Hazra, Harishchander Anandaram

**Affiliations:** 1 Department of Pharmacology, Maharajah’s Institute of Medical Sciences and Hospital, Dr. N. T. R. University of Health Sciences, Dr. Y. S. R. University of Health Sciences, Andhra Pradesh, India; 2 Amrita School of Artificial Intelligence, Coimbatore, Amrita Vishwa Vidyapeetham, Coimbatore, Tamil Nadu, India

**Keywords:** artificial intelligence, drug repurposing, mechanism-aware AI, pharmacovigilance, safety validation, translational pharmacology

Affiliation Amrita School of Artificial Intelligence, Coimbatore, Amrita Vishwa Vidyapeetham, Coimbatore, Tamil Nadu, India was omitted from the paper. This affiliation has now been added for author Harishchander Anandaram.

Affiliation Department of Medicine, College of Nursing, Gulf Medical University, Ajman, United Arab Emirates was erroneously included in the paper. This affiliation has now been removed for author Harishchander Anandaram.

The original version of this article has been updated.

