# Supplementary material for: Mechanism-aware and safety-validated AI for drug repurposing: a critical review and translational pharmacology framework
Source: Front Pharmacol. 2026 Sep 18;17:1915973. doi: 10.3389/fphar.2026.1915973 (PMC13631471; doi:10.3389/fphar.2026.1915973)
Supplement: Supplementary file 1 [file Table1.docx]

# Supplementary Table S1. Database-specific search strategies used for the structured literature search

Database-specific search strategies were adapted according to indexing systems, controlled vocabulary, and available field tags. The final search date was 30 June 2025.

| Database | Platform | Search date | Database-specific search strategy |
| --- | --- | --- | --- |
| PubMed/MEDLINE | PubMed | 30 June 2026 | ("drug repurposing"[Title/Abstract] OR "drug repositioning"[Title/Abstract] OR "drug reprofiling"[Title/Abstract]) AND ("artificial intelligence"[Title/Abstract] OR "machine learning"[Title/Abstract] OR "deep learning"[Title/Abstract] OR "neural network*"[Title/Abstract] OR "knowledge graph*"[Title/Abstract] OR "graph neural network*"[Title/Abstract] OR "network medicine"[Title/Abstract] OR "foundation model*"[Title/Abstract] OR "large language model*"[Title/Abstract]) AND (mechanism*[Title/Abstract] OR pharmacolog*[Title/Abstract] OR "target engagement"[Title/Abstract] OR pathway*[Title/Abstract] OR biomarker*[Title/Abstract] OR ADMET[Title/Abstract] OR toxic*[Title/Abstract] OR safety[Title/Abstract] OR pharmacovigilance[Title/Abstract] OR "drug-drug interaction*"[Title/Abstract] OR "real-world evidence"[Title/Abstract] OR "electronic health record*"[Title/Abstract] OR validation[Title/Abstract]) |
| Scopus | Elsevier Scopus | 30 June 2026 | TITLE-ABS-KEY(("drug repurposing" OR "drug repositioning" OR "drug reprofiling") AND ("artificial intelligence" OR "machine learning" OR "deep learning" OR "neural network*" OR "knowledge graph*" OR "graph neural network*" OR "network medicine" OR "foundation model*" OR "large language model*") AND (mechanism* OR pharmacolog* OR "target engagement" OR pathway* OR biomarker* OR ADMET OR toxic* OR safety OR pharmacovigilance OR "drug-drug interaction*" OR "real-world evidence" OR validation)) |
| Web of Science Core Collection | Clarivate Web of Science | 30 June 2026 | TS=(("drug repurposing" OR "drug repositioning" OR "drug reprofiling") AND ("artificial intelligence" OR "machine learning" OR "deep learning" OR "neural network*" OR "knowledge graph*" OR "graph neural network*" OR "network medicine" OR "foundation model*" OR "large language model*") AND (mechanism* OR pharmacolog* OR "target engagement" OR pathway* OR biomarker* OR ADMET OR toxic* OR safety OR pharmacovigilance OR "drug-drug interaction*" OR "real-world evidence" OR validation)) |
| Embase | Elsevier Embase | 30 June 2026 | ('drug repurposing'/exp OR 'drug repositioning':ti,ab OR 'drug reprofiling':ti,ab) AND ('artificial intelligence'/exp OR 'machine learning'/exp OR 'deep learning':ti,ab OR 'neural network*':ti,ab OR 'knowledge graph*':ti,ab OR 'graph neural network*':ti,ab OR 'network medicine':ti,ab OR 'foundation model*':ti,ab OR 'large language model*':ti,ab) AND (mechanism*:ti,ab OR pharmacolog*:ti,ab OR 'target engagement':ti,ab OR pathway*:ti,ab OR biomarker*:ti,ab OR ADMET:ti,ab OR toxic*:ti,ab OR safety:ti,ab OR pharmacovigilance:ti,ab OR validation:ti,ab) |

## Additional targeted searches

| Topic | Search strategy |
| --- | --- |
| AI reporting and methodological guidance | ("artificial intelligence" OR "machine learning") AND (reporting OR transparency OR reproducibility OR validation OR "TRIPOD-AI" OR "CONSORT-AI") |
| Pharmacovigilance and safety assessment | ("artificial intelligence" OR "machine learning") AND (pharmacovigilance OR "adverse drug reaction*" OR "safety signal*" OR disproportionality) |
| Regulatory guidance | ("artificial intelligence" OR "machine learning") AND (regulatory framework OR medical product OR drug development OR lifecycle) |
| Real-world evidence and validation | ("artificial intelligence" OR "machine learning") AND ("real-world evidence" OR electronic health record* OR registry OR "target trial emulation") |
